# Identification of proteins responsible for adriamycin resistance in breast cancer cells using proteomics analysis

**DOI:** 10.1038/srep09301

**Published:** 2015-03-30

**Authors:** Zhipeng Wang, Shuang Liang, Xin Lian, Lei Liu, Shu Zhao, Qijia Xuan, Li Guo, Hang Liu, Yuguang Yang, Tieying Dong, Yanchen Liu, Zhaoliang Liu, Qingyuan Zhang

**Affiliations:** 1 Department of Medical Oncology, The Third Affiliated Hospital of Harbin Medical University, Haping Road 150 of Nangang District, Harbin 150081, Heilongjiang Province, China; 2 Department of Children's and Adolescent Health, Public Health College of Harbin Medical University, Harbin 150081, Heilongjiang Province, China; 3 College of Bioinformatics Science and Technology, Harbin Medical University, Harbin. 150081, Heilongjiang Province, China; 4 Cancer Research Institute Harbin Medial University, Haping Road 150 of Nangang District, Harbin 150081, Heilongjiang Province, China; 5 Department of Medical Oncology, The Fourth Affiliated Hospital of Harbin Medical University, Harbin, China

## Abstract

Chemoresistance is a poor prognostic factor in breast cancer and is a major obstacle to the successful treatment of patients receiving chemotherapy. However, the precise mechanism of resistance remains unclear. In this study, a pair of breast cancer cell lines, MCF-7 and its adriamycin-resistant counterpart MCF-7/ADR was used to examine resistance-dependent cellular responses and to identify potential therapeutic targets. We applied nanoflow liquid chromatography (nLC) and tandem mass tags (TmT) quantitative mass spectrometry to distinguish the differentially expressed proteins (DEPs) between the two cell lines. Bioinformatics analyses were used to identify functionally active proteins and networks. 80 DEPs were identified with either up- or down-regulation. Basing on the human protein-protein interactions (PPI), we have retrieved the associated functional interaction networks for the DEPs and analyzed the biological functions. Six different signaling pathways and most of the DEPs strongly linked to chemoresistance, invasion, metastasis development, proliferation, and apoptosis. The identified proteins in biological networks served to resistant drug and to select critical candidates for validation analyses by western blot. The glucose-6-phosphate dehydrogenase (G6PD), gamma-glutamyl cyclotransferase (GGCT), isocitrate dehydrogenase 1 (NADP+,soluble)(IDH1), isocitrate dehydrogenase 2 (NADP+,mitochondrial) (IDH2) and glutathione S-transferase pi 1(GSTP1), five of the critical components of GSH pathway, contribute to chemoresistance.

Chemoresistance is a poor prognostic factor in breast cancer, presenting a significant clinical challenge. The resistance to anticancer drugs, such as anthracycline, is still a major cause of chemotherapy failure in cancer patients^1^,^2^.

The anthracycline drug, adriamycin, is one of the most important anti-cancer chemotherapeutic drugs, and is generally used to treat solid tumors and acute leukemias^3^,^4^,^5^,^6^. However, adriamycin resistance has been widely reported in the analysis of cancer research on breast cancer, leukemia, osteosarcoma, lung cancer, etc^7^,^8^,^9^,^10^. A range of factors contributing to this chemoresistance have been proposed, including increased efflux through the P-glycoprotein (P-gp) and multidrug resistance-associated protein (MRP) drug pumps, increased GSH transferases, alterations in topoisomerase activity, and increases in antiapoptotic molecules such as Bcl-2^11^,^12^,^13^,^14^. Potent nuclear DNA repair systems also contribute substantially to the ability of tumor cells to withstand the cytotoxic effects of adriamycin. Despite being occasionally mentioned, to date there have been no studies which fully unravel the expressional signatures of adriamycin resistant breast cancer on the proteomic level. Understanding the mechanisms behind the perspective of proteomic profiling will ultimately facilitate treatments with enhanced tumor responses in the clinic.

To date, proteomic technology has been applied to a wide range of cancer studies including analysis of drug resistance^15^. Mass spectrometry (MS)-based proteomics often involves analyzing complex mixtures of proteins derived from cell or tissue lysates or from body fluids on a global scale^16^,^17^,^18^. In recent years MS-based proteomics has greatly benefitted from enormous advances in high resolution instrumentation. In particular, a novel mass spectrometer, the Q Exactive, couples a mass selective quadrupole to the Orbitrap analyzer, which has been proven to be a popular instrument configuration^19^. In this bench-top instrument, precursor ions are selected by the quadrupole, fragmented by Higher Energy Collisional Dissociation (HCD), and measured at high-resolution and accurate-mass (HR/AM) in the Orbitrap analyzer^20^. Consequently, the Q Exactive offers the potential to analyze many more peptides in a given time, with very high MS/MS data quality^21^. We wanted to combine these benefits with a compact Ultra High Pressure Liquid Chromatography (UHPLC) system, known as the EASY-nLC 1000, which was not available to us in previous single-run analysis. To quantify protein expression changes, we applied TmT labeled samples together with tandem MS to determine differences between the drug sensitive and drug resistant cells. Protein intensities resulted from the average of the single TmT reporter ion intensities^22^.

The aim of this study is to conduct an *in vitro* investigation into adriamycin-resistance mechanisms in breast cancer cells using proteomic strategies, to increase our understanding of the molecular processes involved, and to identify potentially valuable diagnostic or therapeutic resistance biomarkers. To meet these aims, we have run a minimalistic proteomic workflow and presented the quantitative proteomic profiles of two breast cancer cells: MCF-7 and MCF-7/ADR, which are of the same breast cancer origin, but are characterized by different reactions to adriamycin, and therefore, could possibly represent differences in the active molecular networks. The core of this work lies in the identification of cancer up/down regulated proteins externalized by cells, based on the combined exploitation of MS and bioinformatics tools.

## Results

The present study was based on three major sets of experiments. First, the nanoflow liquid chromatography (nLC) and tandem mass tags (TmT) quantitative mass spectrometry were applied to distinguish the DEPs between the MCF-7 and its adriamycin-resistant counterpart, MCF-7/ADR cell lines. Subsequently, western blot analyses of DEPs of GSH metabolism pathway served to validate the results by the new proteomic method. Last, DEPs served to evaluate the potential significance of drug resistance using bioinformatic analysis. These analyses served to assess the potential resistance proteins and novel related proteins in adriamycin-resistant breast cancer.

### Protein profiling

We were able to identify proteins having a wide range of MW, which spanned mainly from 10,000 to 200,000 Da (Supplementary Fig. S1). Most proteins had a pI between 4 and 10 (Supplementary Fig. S1). In this case, to improve analytical precision, all quantitative data associated with a certain peptide that was identified in multiple fractions were used for deriving protein quantitation tables. With this criteria, more than 63,900 MS/MS spectra were matched to peptide sequences and used for protein quantitation in cell proteomics analysis, excluding spectra deriving from contaminants and reversed protein sequences. We obtained more than 9448 peptides, corresponding to 719 proteins which were identified and quantified in the mixed cell sample. Quantitation data were retrieved from MS/MS reporter ions quantifier from PD. More than two unique peptides were considered for each protein quantitation.

### Bioinformatic analysis: identification and functional enrichment of DEPs

**Figure 1:**
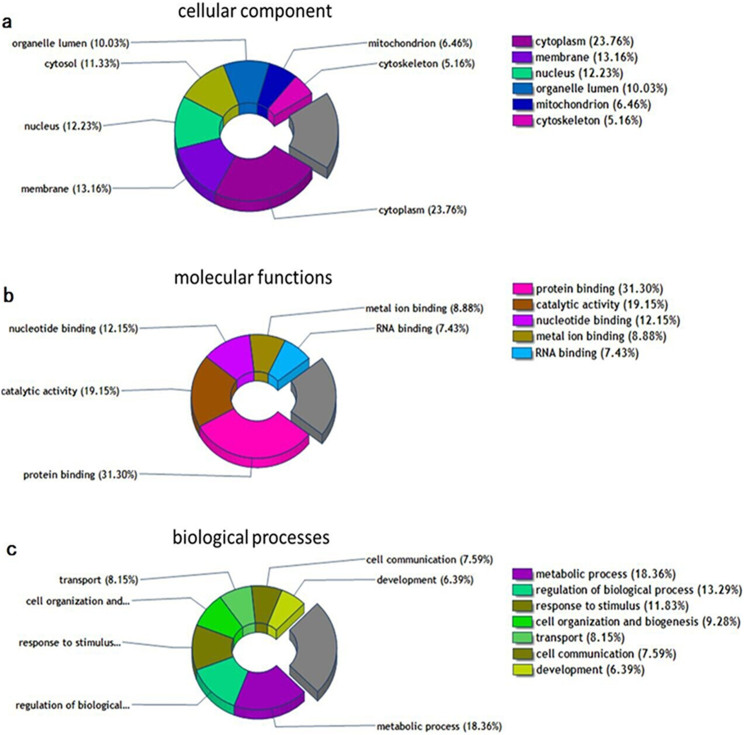
These Venn diagrams demonstrate the GO analyses of all the identified proteins by Proteome Discoverer software. (a) Cellular component analyses of the identified proteins. (b) Molecular functions analyses of the identified proteins. (c) Biological processes analyses of the identified proteins.

Using the DAVID network analysis tool, we analyzed the molecular functions/localizations of the protein data sets according to GO functional annotations and categories. We performed GO analysis on cellular components (CC), molecular functions (MF), and biological processes (BP) of the 719 proteins. A Venn diagram shows the GO analysis of the identified proteins by PD software (Fig. 1). The GO analysis showed that most of the modulated proteins have cytoplasmic origin (23.76%), followed by the membrane region (13.16%), and then nuclear proteins (12.23%) (Fig. 1a). A functional classification of these proteins revealed that most were involved in protein binding, catalytic activity, as well as nucleotide binding and biosynthesis. The remaining molecular functions showed enzyme regulation, signal transduction, etc (Fig. 1b). In Fig. 1c, our BP analysis indicated that the greatest changes occurred in metabolic processes, regulation of biological processes, responses to stimulus, cell organization, and biogenesis. Most of those BP feed into transport, communication, apoptosis, differentiation, and proliferation, suggesting that alterations in these might be involved in adriamycin resistance.

**Figure 2:**
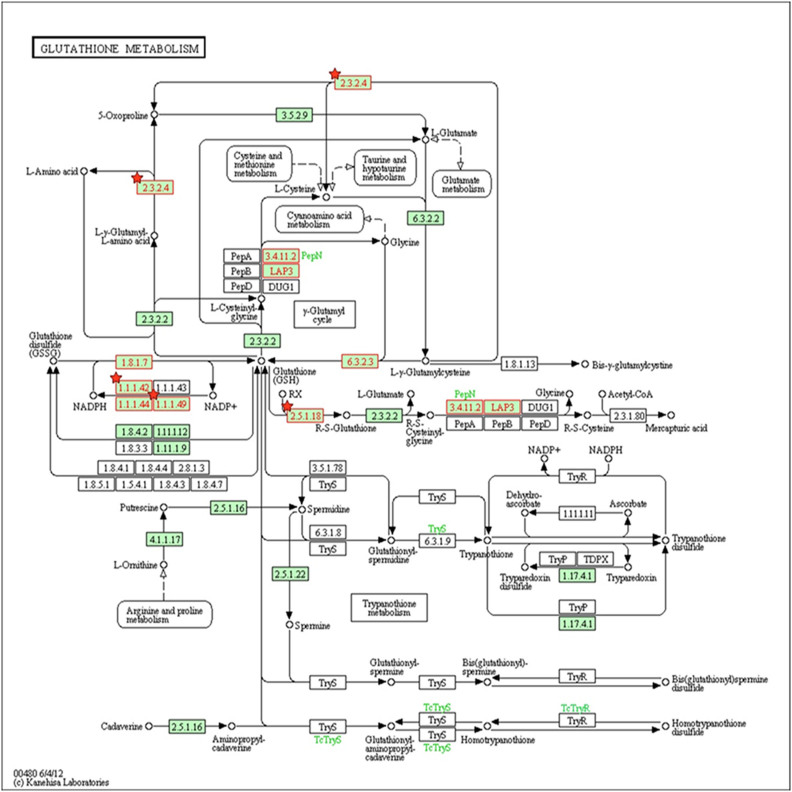
GSH metabolism pathway. Proteins indicated in red were significantly enriched within the GSH metabolism pathway. Red stars represent DEPs. 2.3.2.4.: GGCT; 1.1.1.42: IDH1 and IDH2; 1.1.1.49: G6PD; 2.5.1.18: GSTP1.

Next, as found with KEGG pathway enrichment, the most active pathways are those of endocytosis, spliceosomes, oxidative phosphorylation, GSH metabolism, and the pentose phosphate pathway (PPP) (Supplementary Table S1). Most of the above-mentioned pathways are strictly connected to growth factor response, invasion, motility, and resistance. For instance, 14 proteins were significantly enriched within the GSH metabolism pathway, which is closely related to the chemoresistance (Fig. 2 and Table 1). At 46.5%, coverage of the proteins in the KEGG database it was lower in the cell proteome than the coverage of the three GO categories (CC 83.5%, MF 87.8%, BP 85.7%).

**Figure 3:**
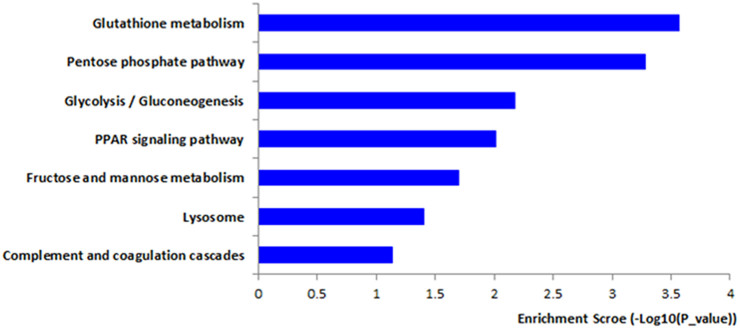
DEPs were enriched within KEGG pathway.

Finally, we focused on the results concerning DEPs exhibiting significant modulation. With a threshold of 1.5 fold-change (≥1.5 or ≤0.67), we found 80 DEPs showing 49 up-regulated and 31 down-regulated proteins between MCF-7 and MCF-7/ADR cells (Supplementary Table S2). When the same analytical method was used, the coverage of the DEPs in the KEGG database was 43% (Supplementary Table S3). KEGG pathway analysis showed that the DEPs were significantly enriched with those related to GSH metabolism, the PPP, glycolysis/gluconeogenesis, the PPAR signaling pathway, etc. (Fig. 3 and Supplementary Table S3). The coverage of the three GO categories was as follows: 89.9% (CC), 86.1% (MF), and 84.8% (BP). In order to better investigate the chemoresistant relevance of the network and to evaluate the property of a single protein, we conducted a further analysis with total protein KEGG as the background and combined the pathways of the DEPs. Those that share the same pathways were shown below: GSH metabolism (Fig. 2), PPP, glycolysis/gluconeogenesis, fructose, and mannose metabolism and the lysosome pathway.

Focusing on the rich degree, the network showed that the main modulated signaling pathways were the GSH metabolism pathway and the PPP. In the GSH metabolism pathway we had found the following 5 DEPs (G6PD, GGCT, IDH1, IDH2, and GSTP1) significantly difference. (Fig. 2 and Table 1). Other proteins were found either increased or decreased in MCF-7/ADR relative to MCF-7 (Table 2). Of note, ABCB1 (also known as MDR1 or P-gp) was found highly up-regulated in our study (Table 2), which is known to be a key player in mediating multidrug resistance (MDR) in cancer. As expected, our analysis also identified up-regulated Annexin A2(ANXA2) and Annexin A5(ANXA5) in the mixed cell sample, which were known to adriamycin resistance (Table 2).

### Bioinformatic analysis: DEPs-one step interacting proteins network in PPI

**Figure 4:**
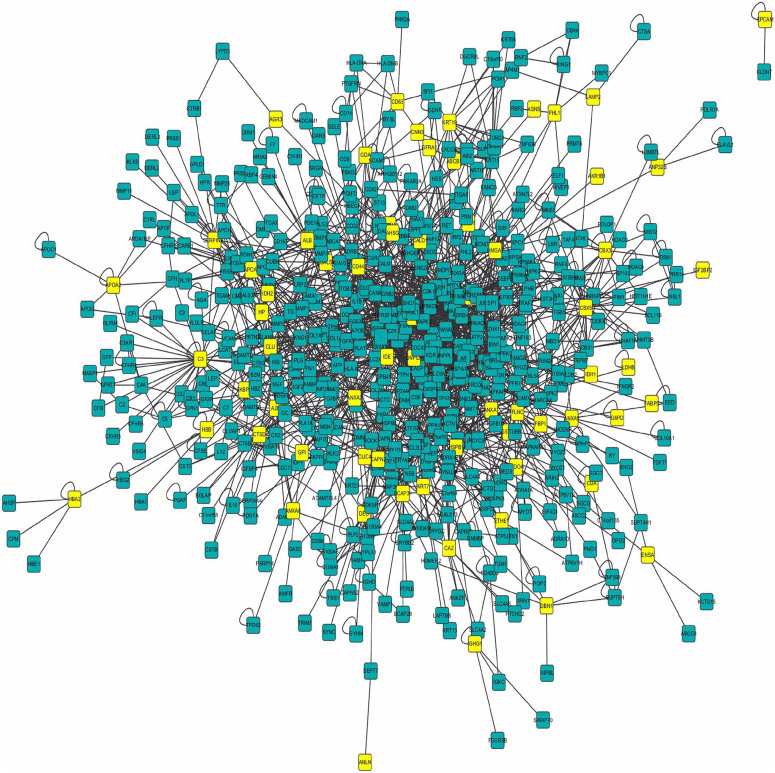
Biological interaction network of DEPs and their one-step interacting proteins. In this network, proteins are represented as nodes, and the biological relationship between two nodes is represented as an edge. The yellow node indicates DEPs and the green node indicates one-step interacting protein. There are 559 nodes and 1899 edges, including 66 DEPs and 493 one-step interacting proteins.

Human protein interacting data sets were downloaded from HPRD, which including 9617 proteins and 39240 interacting relations. In the HPRD database, we found 66 DEPs and used as seed proteins for further network research (Table 3). One-step interacting proteins which were considered to correlate with DEPs tightly were mined and the network was built (Fig. 4). In this network, DEPs and one-step interacting proteins are represented as nodes, and the biological relationship between two nodes is represented as an edge. There are 559 nodes and 1899 edges, including 66 DEPs and 493 one-step interacting proteins. Fig. 4 shows a global view of the DEPs-one step interacting proteins network with the following color-coded nodes and edges: DEPs (yellow), one-step proteins (green). Then, we analyzed the function of DEPs-DEPs and DEPs-one step interacting proteins respectively.

### Bioinformatic analysis: DEPs-DEPs relations

In the network, there are nine proteins which not only DEPs but also one-step interacting proteins (Table 3). They form five pairs of interacting proteins. According to literature, direct relation means that one protein has been reported to be relevant to drug resistance, indirect indicates that one protein has no evidence as resistance, but its one-step protein has drug resistant function, we can regard this protein as the resemble function. Three-pair DEPs (HBB-HBA2, HP-HBB, KRT8-ANXA1) had been reported directly to have association with resistance to drug^23^,^24^,^25^,^26^,^27^,^28^. Another pair ANXA6-A2M, ANXA6 had been proved as a drug resistance protein^29^, so we thought A2M might confer resistance indirectly according to the tightly interaction with ANXA6. Although CBX5-CBX3 had no direct evidence with resistance, they were indirectly verified by their other one-step proteins which had resistant function in the network.

### Bioinformatic analysis: DEPs-one step interacting protein relations

In the network, there are some certain proteins correlated with more than one DEPs, which we thought had closer relation with DEPs and drug resistance. If the one-step protein has been proved having drug resistance function, we can deduce the DEPs have the similar resistant function. An independent analysis of DEPs-one step interacting proteins relations was performed. The result showed that the number of DEPs interacting with one-step protein ranged from 5 to 1 (Supplementary Fig. S3). Biological interactions were shown previously for these proteins (Fig. 4). These one-step proteins which correlated with more than one DEPs should be focused on, for they might be highly correlate with adriamycin resistance and could be further study.

### Literature validation

From literature, we collected scientific publications of DEPs and one-step interacting proteins that have been experimentally discovered and verified. We made further correction by two professors about the text mining results on the DEPs (Table 4). 64(97%) DEPs had been reported to have correlation with drug resistance. Among them, 49(76.6%) DEPs had been proved as drug resistance proteins directly, 15(23.4%) DEPs were verified indirectly. Only 2(3%) DEPs had no evidence to demonstrate the correlation with resistance (Table 4). These results were highly in accordance with the proteomics data, which further validate the accuracy of our proteomic experimental method.

### Bioinformatic analysis: Modules Identification in Networks

To further discover the relationship of the DEPs, we dig out 32 modules in the network (Supplementary Table S4). Such potentially biological relevant associations are inferred from either direct or indirect (including intra- and inter-module) interactions. Among these modules, we randomly took one module which contained 20 proteins for further research (Supplementary Fig. S2). The 20 proteins were enriched within 11 pathways, among them, there were six pathways associated with resistance, especially MAPK signal pathway^30^. There were five proteins in MAPK signal pathway, including one pair of DEPs-one step interacting protein (HSPB1-DAXX).

### Validation by western blot on DEPs in GSH pathway

**Figure 5:**
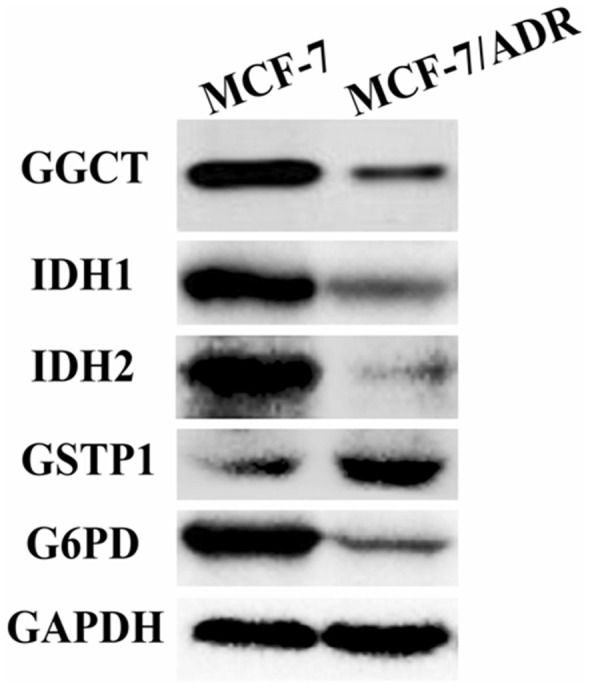
Western blot analysis confirmed changes in protein expression initially identified by quantitative proteomics method. The expression of DEPs was focused on GSH metabolism pathway. G6PD, GGCT, IDH1 and IDH2 were found down–regulated in MCF-7/ADR cells compared with MCF-7 cells. Up-regulated expression was observed for GSTP1. GAPDH was used as the loading control.

The expression of G6PD, GGCT, IDH1, IDH2 and GSTP1 were further validated by western blot. Consistent with the observations in proteomics analysis, G6PD, GGCT, IDH1 and IDH2 were found down-regulated in MCF-7/ADR cells compared with MCF-7 cells, and GSTP1 was found up-regulated in MCF-7/ADR cells compared with MCF-7 cells (Fig. 5).

## Discussion

Quantitative proteomics is driving the discovery of disease-specific targets and biomarkers^31^. UHPLC, mass spectrometry-based proteomics (Q-Exactive), combined with TmT labeled samples were applied to quantify protein expression changes due to its faster separation, greater sensitivity and resolution.

The novelty of our study deals with the application of this proposed quantitative proteomic approach to dissect the DEPs association with adriamycin-resistance mechanisms in breast cancer cells. This proteomics technique is a powerful method to reach very large coverage of the cell proteome and allow systems wide analysis, and to discovery of DEPs aiming to address potentially valuable diagnostic or therapeutic resistance biomarkers, which may led to a better characterization of the MCF-7/ADR cell line and to a better understanding of its adriamycin resistant phenotype. 80 DEPs were found to be differentially expressed in MCF-7/ADR cells compared with MCF-7 cells, five DEPs (G6PD, GGCT, IDH1, IDH2 and GSTP1) belonging to the GSH pathway were identified significantly different. To validate results of the proteins identified in the proteomics experiments, western blot analyses were performed on these 5 DEPs in MCF7 and MCF-7/ADR cells (Fig. 5).

**Figure 6:**
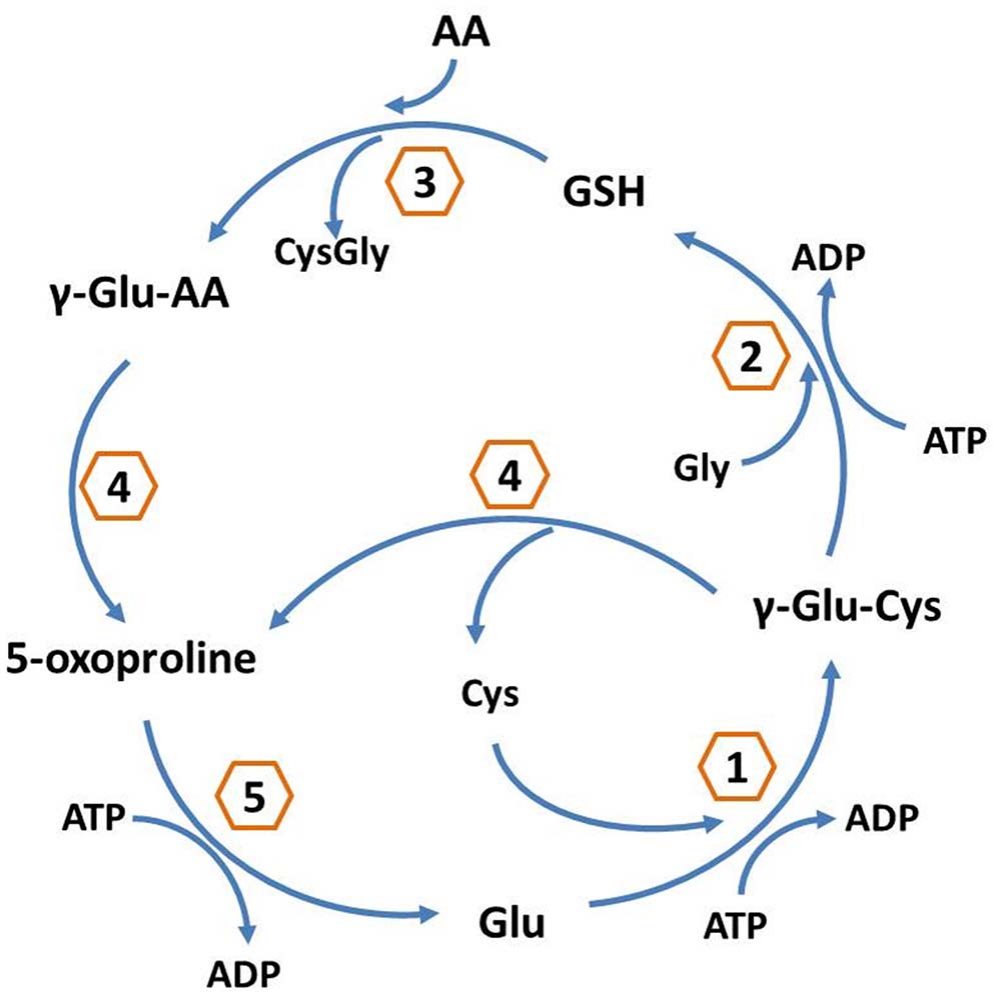
Enzyme-catalyzed reactions in the γ-glutamyl cycle. 1: γ-glutamyl cysteine synthetase; 2: glutathione synthetase; 3: γ-glutamyl transpeptidase; 4: GGCT; 5: 5-oxoprolinase.

The GSH metabolism pathway contributes to the detoxification and elimination of a wide range of xenobiotic compounds, underscoring the role of redox regulation of MDR mediated by drug efflux pumps^32^,^33^,^34^. In several previous studies GGCT, a critical component of the GSH pathway, has been implicated as a cancer marker with a potential role in cell proliferation. Despite the differential expression of GGCT in tumor tissues^35^,^36^,^37^, little is known about the function of GGCT in cancer resistant cells. The γ-glutamyl cycle is a pathway that encompasses the synthesis and degradation of GSH and is thought to contribute to the uptake of amino acids across cellular membranes^38^. GGCT is a pivotal enzyme that contributes to the γ-glutamyl cycle regulating GSH metabolism through catalyzing the formation of 5-oxoproline (pyroglutamic acid) from γ-glutamyl dipeptides^34^,^39^. Aaron J Oakley *et al.* reported^39^ that the inhibition of GGCT in cases of GSH synthetase deficiency blocks the degradation of γ-glutamylcysteine and allows it to accumulate to a level where it may partially substitute for GSH in redox and detoxification reactions (Fig. 6). There is a significant turnover of GSH and γ-glutamyl-amino acid dipeptides via GGCT and the γ-glutamyl cycle under normal metabolic conditions (Fig. 2 and Fig. 6). The position of GGCT in the γ-glutamyl cycle suggests that it could play a significant role in regulating the synthesis of GSH by limiting the availability of γ-glutamylcysteine. Aaron J Oakley *et al*.^39^ also discuss the feedback inhibition of γ-glutamyl cysteine synthetase by GSH. Based on this process, we further hypothesize that GSH expression is modulated by GGCT dependent negative feedback. In our analysis GGCT was found down-regulated in MCF-7/ADR cells (Table 2), while GSH synthetase was not found statistically regulated in the mixed samples. Moreover, the expression of GGCT was also independently shown by observations from western blot analyses, the expression of GGCT was down-regulated in MCF-7/ADR cells compared with MCF-7 cells which supporting proteomic results (Fig. 5). These results suggest that the decreased activity of GGCT is necessary for MCF-7/ADR cells to maintain a high level of GSH, which in turn exports adriamycin out of the cell. Our data provide a novel mechanism for the acquisition of adriamycin resistance.

An alternative and less investigated way to increase the level of GSH is to activate the PPP flux via the activation of G6PD^33^. As the earlier workers have pointed out, G6PD catalyses the first and rate-limiting step of the PPP and a major source of NADPH. NADPH is used by GSH reductase to reduce glutathione disulfide (GSSG) to GSH^33^. P. Manuela *et al.*^33^ showed the increase expression of G6PD in the adriamycin-resistant human colon cancer cellline HT29-DX when compared with normal HT29 cells. Although evidence has been provided that oxidative stress plays a role in resistant cells, many controversial data persist. As demonstrated in another study^40^ as well as in our study(Table 1), the decrease in the expression of G6PD in MCF-7/ADR was surprisingly found, probably due to the different cell types investigated. Western blot were performed for G6PD to further validate the accuracy, the expression of G6PD was down-regulated in MCF-7/ADR cells compared with MCF-7 cells. This discrepancy suggests a further mechanism study for GSH increase and its effects on MDR, i.e. the hyperactivity of the PPP and of its rate-limiting enzyme G6PD. IDH1 and IDH2 are NADP+ dependent enzymes that catalyze the oxidative decarboxylation of isocitrate to α-ketoglutarate (α-KG), generating NADPH from NADP+^41^. Somatic heterozygous mutations in *IDH1* and *IDH2* have been identified in a number of cancers^42^,^43^,^44^. There is increasing evidence that the prognostic impact of *IDH1*and *IDH2* mutations varies according to the specific mutation and also depends on the context of concurrent mutations of other genes^41^. In our study, we found the expression of IDH1 and IDH2 was down-regulated in MCF-7/ADR cells (Table 2), we do found that the expression of IDH1 and IDH2 down-regulated by western bolt in MCF-7/ADR cells compared with MCF-7 cells (Fig. 5), which is in good agreement with the proteomic data. Our data suggested G6PD, IDH1 and IDH2 may carry out a novel mechanism with adriamycin resistance in breast cancer.

In addition to the differentially expressed proteins discussed above, several other proteins were found to have remarkably altered expression. A number of drugs including adriamycin are known to be substrates of Glutathione S-transferase (GST) and it has been clearly shown that the overexpression of GST and high levels of GSH in tumors are linked to the development of MDR^45^,^46^,^47^. One member of the GST family, GSTP1 up-regulation was identified to be associated with adriamycin resistance in our proteomic experiments (Table 2). Western blot analysis showed the up-regulated expression of GSTP1 in MCF-7/ADR cells compared with MCF-7 cells (Fig. 5), which in accordance with the proteomic result. In addition to the GST family, the most striking up-regulated proteins were HMGA1 in DEPs (Table 2). HMGA1 belongs to the high mobility group A (HMGA) family,which has been previously implicated in breast carcinogenesis^48^. D Angelo D^49^ reported that the blockage of HMGA1 expression was a promising approach to enhance cancer cell chemosensitivity, which supported HMGA1 could increase the sensitivity of cancer cells to antineoplastic drugs. To the best of our knowledge, no previous reports have revealed that HMGA1 is increased in adriamycin-resistant breast cancer. MCF-7/ADR cells also have markedly increased expression of the L-lactate dehydrogenase B chain (LDHB) relative to MCF-7 cells (Table 2), which support the hypothesis that LDHB is a predictive marker for the response for patients with breast cancer receiving neoadjuvant chemotherapy^50^. Furthermore, our proteomic analysis revealed a large number of proteins that may cause cells to adopt an apoptosis-resistant state. For example, the small stress HSP beta-1 (HSPB1 or Hsp27) is well described to counteract apoptosis and its elevated expression is associated with increased aggressiveness of several primary tumors^51^. R. Kanagasabai^52^ reported that the expression of HSPB1 sensitizes MCF-7/ADR cells to adriamycin. But recent reports show that HSPB1 up-regulation can worsen the prognosis of breast cancer and the sensitivity of tumors to chemotherapy and radiotherapy^53^,^54^. HSPB1 implications in cancer cell resistance to adriamycin has been debatable. In our study HSPB1 was found down-regulated (Table 2), which requires further experiments to confirm this result. Several studies have suggested that the acquisition of the MDR phenotype is associated with elevated invasion and metastasis of tumors^55^,^56^.

Overall, western blot experiments were served to provide confidence in the proteomics methodology applied. The expression of G6PD, GGCT, IDH1, IDH2 were down-regulated in MCF-7/ADR cells compared with MCF-7 cells, and GSTP1 was found up-regulated in MCF-7/ADR cells compared with MCF-7 cells. The results obtained are in good agreement with the proteomic method, which strengthen the evidence that proteomic method used in our study is powerful in identification of pathways of drug resistance proteins.

It is important to point out that in our experimental design bioinformatic analysis was carried out to further validate the results obtained from this proposed proteomic method. We use the 66 DEPs called seed proteins and proteins which have direct interactions with DEPs in one step called one-step proteins to build the network map (Fig. 4). If the one-step protein was proved to have drug resistance function, we could deduce the DEPs–one step proteins had the similar resistant function. In the network, the relation of DEPs-DEPs was investigated at high priority. Nine DEPs formed five paires of DEPs-DEPs, among them, six DEPs had been proved directly as drug resistance-associated protein, three DEPs were also deduced to relate with resistance indirectly. An independent analysis of DEPs-one step interacting proteins relations was performed. SUMO4 as a one-step interacting protein was reported to resistant with Type 1 diabetes mellitus^57^, which was referred to five DEPs (GSTP1, G6PD, IDH1, ALDOA, LDHB) simultaneously. These five proteins had been proved to be correlated with resistant drug directly or indirectly by HPRD. We also obtained that there were four one-step proteins correlate with four DEPs respectively, nine one-step proteins correlate with three DEPs respectively, 63 one-step proteins correlate with two DEPs respectively and 425 one-step proteins correlate with one DEPs in the network (Supplementary Table S5,Fig. S3). From literature, we collected scientific publications of DEPs and one-step interacting proteins that have been experimentally discovered and verified. We aimed at these high credible proteins and make further manual correction. 97% DEPs in the HPRD database had been reported to have correlation with resistant drug. To further discover the relationship of the DEPs, we dig out 32 modules in the network. The 2^nd^ module was focused on further research, which including 20 proteins enriched within 11 pathways, among them, MAPK signal pathway was involved in resistance^30^, which enriched five proteins in network, including one pair of DEPs-one step interacting protein (HSPB1-DAXX). These results were highly in accordance with the proteomics data, which further validate the accuracy and feasibility of our proteomic experimental method, and support the results from proteomics.

In order to guarantee the data source of built network accuracy, we chose HPRD, a strictly protein interacting database which had been experimentally discovered and verified, rather than predicting proteins interacting database. 66 DEPs involved in HPRD, which led to experimental data couldn't be totally validated. For example, GGCT aforementioned validation by western blot was not existed in HPRD, so the bioinformatic result didn't cover GGCT. One reason is that HPRD may be not a whole protein interacting data and need to be updated timely. Another cause is GGCT may be a new resistant drug protein. It is the first time to find the new resistant function of GGCT by our study. Moreover, experimental testing of these hypotheses will be required to support further assessments for potential clinical application.

Despite the pilot study presented here, two further questions require in depth studies. Firstly, are any of the elements in this proteomic profile predictive of clinical responses to adriamycin? On-going research to correlate *in vivo* protein expression signatures and clinical responses will address this issue. Secondly, are the aberrant proteins identified central to the mechanism of cellular adriamycin resistance? Functional assays that relate differential expression of these novel proteins to the generation or maintenance of the drug-resistant phenotype will improve our understanding of the roles of these proteins in cancer drug resistance.

## Conclusions

Although still in its infancy, the use of proteomics is an excellent approach for the discovery of predictors that can be used for individualization of treatment for breast cancer patients. Our proteomic studies in breast cancer cells have revealed a number of promising proteins that might serve as candidate biomarkers of prognosis and chemotherapy. Hopefully, these findings can be exploited in the future as a useful source of information to guide targeted experiments aiming at discovering yet unknown chemoresistant mechanisms and therapeutic strategies.

## Methods

### Chemicals and reagents

TmT duplex isobaric tags reagent set (TMT^2^) and trypsin for MS were purchased from Thermo Scientific (San Jose, CA, USA). EDTA-free protease inhibitor cocktail was purchased from Roche Diagnostics (Indianapolis, IN, USA). Phosphatase inhibitors were purchased from Pierce (Idaho, ID, USA). RIPA Lysis Buffer was purchased from Thermo Scientific (San Jose, CA, USA). The Bradford assay was purchased from Applygen (Beijing, China). C18 ZipTip micropipette tips were purchased from Millipore (Bedford, MA, USA). Generic chemicals were purchased from Sigma-Aldrich (St. Louis, USA). All of the chemicals and reagents used in this study were of analytical chromatography grade.

### Cell culture

The breast cancer cell line MCF-7 and its adriamycin resistant counterpart MCF-7/ADR were purchased from the Cancer Institute & Hospital (CIH), Chinese Academy of Medical Sciences (CAMS). Cells were cultured in RPMI 1640 (Gibco, Invitrogen, Carlsbad, CA, USA) containing 10% fetal bovine serum (Hyclone), 2 mM L-glutamine, streptomycin, 100 IU/mL penicillin (all from Gibco-Invitrogen Corp., UK) and maintained without/with 0.2 μg/ml ~ 0.5 μg/ml adriamycin, respectively. All cells were incubated at 37°C in a humidified atmosphere containing 5% CO2. The cells were passaged every 2 ~ 3 days.

### Sample preparation

Cells were cultured to harvest 100 μg of protein per sample according to the TmT manufacturer's instructions. 45 ul of 200 mM triethyl ammonium bicarbonate (TEAB) was added to the each protein sample and followed with 5 ul of 2% SDS and 5 ul of 200 mM tris (2-chloroethyl) phosphate (TCEP). The reaction was incubated for 1 hour at 55°C. According to the manufacturer's instructions, 5 ul of 375 mM iodoacetamide (IAA) was added for 30 min at room temperature in the dark.

The protein was then precipitated overnight in six volumes of pre-chilled (−20°C) acetone. In order to improve protein identification and characterization, 4 μL (1 μg/μL) of trypsin was added per sample, and the digestion was performed at 37°C for 12 hours. Finally, enzymatic digestion was performed by adding 2 μL (1 μg/μL) of trypsin to the sample and incubating it at 37°C for 6 hours.

The reporter ions are characteristic of each tag form and detected at distinct m/z (i.e., 126–127 Da for TMT^2^). These reporter ions are in the low mass region of the MS/MS spectrum and are used to report relative protein expression levels during peptide fragmentation. Peptide samples from MCF-7 were labeled with TMT^2^-126 isobaric tag according to the manufacturer's protocol, and peptide samples from MCF-7/ADR were labeled by adding the same amount of TMT^2^-127 isobaric tag. Two pools of labeled peptide samples were combined at equal amount. The dried peptides in the mixed cell sample were cleaned and desalted using C18 ZipTip micropipette tips following the manufacturer's user guide.

### Chromatographic and mass spectrometric analysis

All separations were performed on a 150 × 0.050 mm capillary reversed-phase column packed with C18 packing material at room temperature using a Thermo Scientific EASY-nLC1000™ system and a binary solvent system composed of water containing buffer A (0.1% formic acid and 2% acetonitrile) and buffer B (acetonitrile containing 0.1% formic acid). The peptides were separated by a linear gradient of buffer B up to 40% in 200 minutes for a 4 hours gradient run with a flow rate of 300 nl/min in the EASY-nLC 1000 system.

The samples were analyzed with a Thermo Scientific Q Exactive™ hybrid quadrupole-Orbitrap mass spectrometer. The UHPLC was coupled to a Q Exactive mass spectrometer via the nanoelectrospray source (Thermo Fisher Scientific). The Q Exactive was operated in data dependent (dd) mode with full scans acquired at a resolution of 70,000 at 350 m/z and with dd-MS/MS scans acquired at a resolution of 17,500. The mass spectrometer was operated in positive mode in the scan range of 350 ~ 2,000 m/z. Fixed first m/z is 100 in dd-MS/MS scans. Up to the top 15 most abundant isotope patterns with a charge ≥2 from the survey scan were selected with an isolation window of 2.0 m/z. The maximum ion injection times for the full scan and the dd-MS/MS scans were 20 ms and 100 ms respectively, and the automatic gain control (AGC) for the full scan and the dd-MS/MS scans were 3E6 and 1E5 respectively. Repeat sequencing of peptides was kept to a minimum by dynamic exclusion of the sequenced peptides for 30 s.

### Protein identification

We used Proteome Discoverer™ (PD) software version 1.3 (Thermo Scientific) to perform the quantitative proteomic analysis. The MS/MS spectra search was performed by SEQUEST® engines to search against the Uniprot *Homo sapiens* database (http://www.uniprot.org), coupled to the appropriate statistical and quantitative validation methods. Starting with the raw data, PD calculated the relative intensities of reporter ions from a specific identified tandem mass spectrum. A threshold intensity rate of 10,000 for the sum of the reporter ion intensities of HCD spectra was applied. A first statistical evaluation of these large data sets was performed by computing the distribution of m/z measurements. We evaluated analysis performance in terms of molecular weight (MW) and isoelectric point (pI) range of detected proteins. Data derived from MS analysis were examined using percolator for false discovery rate (FDR) < 0.01, which was calculated on the basis of the number of peptide matches against a decoy database. We then performed MS analysis of the TmT labeled samples on Q Exactive. Protein intensities resulted from the average of the single TmT reporter ion intensities obtained for each peptide associated with a specific protein. The average ratio of differential TmT 127/126 expression (1.5 fold increase or decrease) represents the ratio of two samples. In other words, we identified DEPs in our TmT experiment using 1.5 and 0.67 as the up-regulation and down-regulation cutoff points.

### Bioinformatic analysis of the detected proteome

The data sets have been analyzed using bioinformatic methods, extracting information about activated pathways and biomarkers linked to chemoresistance. Specific bioinformatics tools like Database for Annotation,Visualization, and Integrated Discovery (DAVID 6.7) have been used to identify protein and molecular pathway modifications. Gene IDs and gene symbol, corresponding to each protein, were obtained by PD. We imported the list of modulated genes, corresponding to total protein, integrated with the list of singleton proteins into DAVID. To understand high-level functions and utilization of the biological systems from molecular-level information, especially large-scale molecular datasets generated by MS, we utilized the Kyoto Encyclopedia of Genes and Genomes (KEGG) pathway representing our knowledge about molecular interactions and reaction networks. Distributions in subcellular locations and molecular functions were assigned to each protein based on Gene Ontology (GO) categories. The significantly (p < 0.05 and FDR < 0.05) enriched categories are presented here.

Human protein interacting Data were downloaded from HPRD database (http://www.hprd.org/). We use the DEPs and proteins which have direct interactions with DEPs in PPI to build the network map. We then analyzed the topological characteristics, mined modules using MINE^58^, a plugin of Cytoscape^59^, and identified associations between modules and KEGG biological processes using DAVID. We examined the Web of Knowledge and NCBI PubMed databases with the keywords “resistant” and gene symbol from the abstracts. From these articles, we manually extracted DEPs, which have been screened by HPRD for drug resistance. Although the number of DEPs collected by the literature search is limited, they are highly trustworthy and thus they lay the foundation for our results.

### Western blot analysis

In order to validate data obtained from proteomics analysis, the expression of DEPs which focused on GSH metabolism pathway have been determined by western blot analysis, including G6PD, GGCT, IDH1, IDH2 and GSTP1. MCF-7 and MCF-7/ADR cell proteins were extracted in RIPA lysis buffer and quantified by the Bradford assay. Samples were separated on 12% SDS–PAGE and transferred to a PVDF membrane. The membranes were blocked with 5% nonfat dry milk for 2 h at room temperature and subsequently probed with the primary antibodies: rabbit-anti-G6PD polyclonal antibody (diluted 1:500 ABclonal, China), rabbit- anti-GGCT polyclonal antibody (diluted 1:400 Proteintech, China), rabbit-anti-IDH1 polyclonal antibody (diluted 1:500 ABclonal, China), rabbit-anti-IDH2 polyclonal antibody (diluted 1:500 ABclonal, China), rabbit-anti-GSTP1polyclonal antibody (diluted 1:500 ABclonal, China). Membranes were then probed by incubation with a anti-rabbit secondary antibody (diluted 1:5000) for 1 h at room temperature, conjugated to fluorophores from Rockland Immunochemicals (Gilbertsville, PA). The fluorescent signals were visualized using the Odyssey imaging system (Li-COR, Lincoln, NE). All western blot analyses were repeated at least three times.

## Author Contributions

Z.W., S.L. and Q.Z. conceived the idea and designed the experiments. Performed the experiments: Z.W., S.L., X.L., S.Z., Q.X., L.G. and Y.L. Participated in discussing the results: H.L., Y.Y. and T.D. Analyzed the data: Z.W., S.L. and L.L. Wrote the paper: Z.W. and S.L. Proofread the manuscript: Z.L. and Q.Z. All authors reviewed the manuscript.

## Supplementary Material

Supplementary Information Identification of proteins responsible for adriamycin resistance in breast cancer cells using proteomics analysis

## Figures and Tables

**Table 1:** Fourteen proteins were significantly enriched within the GSH metabolism pathway

Uniprot accession no.	Gene IDs	Gene symbol	Protein description	Coverage^A^	127/126^B^
P15144	290	ANPEP	Aminopeptidase	3.62	1.257
Q9Y2Q3-3	373156	GSTK1	Glutathione S-transferase kappa 1	15.89	0.885
P11413	2539	G6PD	Glucose-6-phosphate 1-dehydrogenase	31.46	*0.498*
P21266	2947	GSTM3	Glutathione S-transferase Mu 3	51.56	0.723
P78417	9446	GSTO1	Glutathione S-transferase omega-1	36.10	1.268
P00390-5	2936	GSR	Glutathione reductase	8.18	0.851
O75223	79017	GGCT	Gamma-glutamylcyclotransferase	21.28	*0.631*
P48637	2937	GSS	Glutathione synthetase	5.27	0.932
O75874	3417	IDH1	isocitrate dehydrogenase 1 (NADP+), soluble	28.50	*0.568*
P48735	3418	IDH2	isocitrate dehydrogenase 2 (NADP+), mitochondrial	40.49	*0.643*
P28838-2	51056	LAP3	Cytosol aminopeptidase	33.20	0.777
P09211	2950	GSTP1	Glutathione S-transferase P	52.86	*1.612*
P10620-2	4257	MGST1	microsomal glutathione S-transferase 1	28.74	0.932
P52209	5226	PGD	6-phosphogluconate dehydrogenase	28.99	0.703

^A^Data associated with the particular peptide from our proteomics analysis were matched to the peptide database.

^B^MCF-7 peptide samples of were labeled with TMT^2^-126 isobaric tags; MCF-7/ADR peptide samples of were labeled with TMT^2^-127 isobaric tags. The values of 127/126 represent the relative quantitation ratio of the DEPs in two cells.

**Table 2:** Some DEPs were identified in MCF-7/ADR compared to MCF-7

Uniprot accession no.	Gene IDs	Gene symbol	Protein description	Coverage A	127/126^B^	Molecular Function	Biological Processes
P08727	3880	KRT19	Keratin, type I cytoskeletal 19	51.75	0.414	protein coding	response to stimuli; cell differentiation;
P13473	3920	LAMP2	lysosome-associated membrane glycoprotein 2	4.88	0.431	protein coding	transport; coagulation; response to stimuli
P11413	2539	G6PD	Glucose-6-phosphate 1-dehydrogenase	31.46	0.498	catalytic activity; protein coding	metabolic processes; regulation of biological processes;
P05787	3856	KRT8	Keratin, type II cytoskeletal 8	56.52	0.520	catalytic activity; protein coding	cell death; cell organization and biogenesis;
P04792	3315	HSPB1	Heat shock protein beta-1	43.90	0.551	enzyme regulator activity; protein coding	development; metabolic processes;
O75874	3417	IDH1	Isocitrate dehydrogenase [NADP] cytoplasmic	28.50	0.568	catalytic activity; protein binding	metabolic processes; response to stimuli;
O75223	79017	GGCT	Gamma-glutamylcyclotransferase	21.28	0.631	catalytic activity; protein binding	regulation of biological processes; metabolic processes
P48735	3418	IDH2	Isocitrate dehydrogenase [NADP], mitochondrial	40.49	0.643	catalytic activity; nucleotide binding	metabolic processes
P08133-2	309	ANXA6	Isoform 2 of Annexin A6	33.39	1.501	metal ion binding; protein binding	transport; regulation of biological processes
P04083	301	ANXA1	Annexin A1	60.69	1.501	DNA binding; enzyme regulator activity;	defense response; regulation of biological processes;
P08758	308	ANXA5	Annexin A5	71.25	1.554	enzyme regulator activity; metal ion binding;	cell communication; regulation of biological processes;
P09211	2950	GSTP1	Glutathione S-transferase P	52.86	1.612	catalytic activity; protein binding;	response to stimuli; defense response;
P07355	302	ANXA2	Annexin A2	56.05	1.907	enzyme regulator activity; metal ion binding;	development; metabolic processes;
P08183	5243	ABCB1	Multidrug resistance protein 1	26.33	1.915	transporter activity; protein binding;	metabolic processes; transport;
Q01469	2171	FABP5	Fatty acid-binding protein	64.44	2.122	transporter activity; protein binding	metabolic processes; transport;
P07195	3945	LDHB	L-lactate dehydrogenase B chain	40.12	2.311	catalytic activity; protein coding	metabolic processes
P17096-2	3159	HMGA1	Isoform HMG-Y of High mobility group protein HMG-I/HMG-Y	17.71	3.484	DNA binding; catalytic activity; protein coding	metabolic processes; response to stimuli;

^A^Data associated with the particular peptides from our proteomics analysis were matched to the peptide database.

^B^MCF-7 peptide samples were labeled with TMT^2^-126 isobaric tags; MCF-7/ADR peptide samples were labeled with TMT^2^-127 isobaric tags. The values of 127/126 represent the relative quantitation ratio of the DEPs in two cells.

**Table 3:** DEPs information obtained from HPRD

DEPs identified by Proteomic method	number	Gene symbol
seed proteins in HPRD	9	HBB, HP, ANXA6, HBA2, KRT8, A2M, CBX3, CBX5, ANXA1
	57	GSTP1, FHL1, APOA2, ANXA2, CLIC4, DNPEP, LGALS3, G6PD, ALB, BCAP31, SERPINA1, ABCB1, ASNS, ANP32B, HSPB1, C3, IGF2BP2, AKAP12, AKR1B1, IGHG1, LAMP2, IDH1, AGR3, GPI, FLNC, CTSD, ALDOA, ANXA5, ENSA, CA2, HMGA1, CD44, GDA, CAPN2, CALD1, FKBP10, CLU, CD63, DBN1, APOA1, HMGN1, ANLN, FABP5, LDHB, EPCAM, FBP1, KRT7, KRT19, AHSG, GFRA1, IDE, CDA, IDH2, CNN3, LIMA1, DES, ETHE1
non-seed proteins in HPRD	14	HIST1H1D, IGLC2, ISOC1, IGHA1, GGCT, APOC3, LXN, KIAA1324, AKR1C4, FSTL1, L1RE1, CMBL, CTSZ, LY6K

**Table 4:** The relation of DEPs-one step interacting proteins were identified by HPRD

	Correlation with drug resistance	No correlation with drug resistance
DEPs	64 (97%)	2(3%)
Direct correlation	49 (76.6%)	-
Indirect correlation	15 (23.4%)	-
