# Supplementary material for: Identification of proteins responsible for adriamycin resistance in breast cancer cells using proteomics analysis
Source: Sci Rep. 2015 Mar 30;5:9301. doi: 10.1038/srep09301 (PMC4377623; doi:10.1038/srep09301)
Supplement: Supplementary Information — Identification of proteins responsible for adriamycin resistance in breast cancer cells using proteomics analysis [file srep09301-s1.pdf]

Identification of proteins responsible for adriamycin resistance in breast cancer cells using proteomics analysis

Zhipeng Wang [1], Shuang Liang [2], Xin Lian [1], Lei Liu [3], Shu Zhao [1], Qijia Xuan [1], Li Guo [1], Hang Liu [1], Yuguang Yang [1], Tieying Dong [1], Yanchen Liu [1], Zhaoliang Liu [4]& Qingyuan Zhang [1]*

Table S1 | The active pathways were found with KEGG pathway enrichment.

Table S2 | Eighty differentially expressed proteins were identified in MCF-7/ADR compared to MCF-7.

Table S3 | DEPS were enriched within KEGG pathway.

Table S4 | Thirty-two modules were identified in network.

Table S5 | Relation of DEPs-one step interacting proteins.

Figure S1 | The molecular weights (MW) of the identified proteins spanned mainly from 10,000 to 200,000 Da. Most proteins had an isoelectric point (pI) between 4 and 10.

Figure S2 | Functional network of identified the 2nd module. This module contained 20 proteins which were enriched within 11 pathways, among them, there were six pathways associated with resistance, especially MAPK signal pathway. There were five proteins in MAPK signal pathway, including one pair of DEPs-one step interacting protein (HSPB1-DAXX).The yellow node indicates DEPs and the green node indicates one-step interacting protein.

Figure S3丨DEPs-one step interacting protein relations. The number of DEPs interacting with one-step protein ranged from 1 to 5. It represented that 425 one-step proteins correlate with 1 DEPs, 63 one-step proteins correlate with 2 DEPs, 9 one-step proteins correlate with 3 DEPs, 4 one-step proteins correlate with 4 DEPs and 1 one-step protein correlate with 5 DEPs in the PPI.

Table S1｜The active pathways were found with KEGG pathway enrichment.

Category	Term	Count	%	PValue	Genes	FDR
KEGG_PATHWAY	hsa05016:Huntington's disease	28	4.057971014	1.68E-05	1350, 10540, 539, 4513, 1347, 515, 1173, 506, 9377, 509, 1329, 581, 293, 1327, 292, 498, 522, 161, 54205, 163, 6389, 1639, 1211, 10476, 1212, 160, 1213, 3065	0.020231
KEGG_PATHWAY	hsa05010:Alzheimer's disease	23	3.333333333	4.88E-04	824, 506, 9377, 1350, 823, 509, 1329, 1327, 3028, 498, 488, 522, 54205, 6389, 10476, 2597, 4513, 539, 808, 3416, 1347, 515, 5594	0.585945
KEGG_PATHWAY	hsa04144:Endocytosis	23	3.333333333	0.002474	3304, 10059, 27243, 128866, 382, 1785, 3312, 25978, 29082, 161, 163, 84364, 1211, 10938, 1212, 160, 1213, 3107, 30844, 3310, 1173, 998, 9146	2.940087
KEGG_PATHWAY	hsa04810:Regulation of actin cytoskeleton	21	3.043478261	0.049101	2934, 55845, 10672, 1073, 1072, 10093, 7430, 10092, 3688, 1399, 10552, 23191, 8826, 10095, 10094, 81, 60, 10109, 87, 998, 5594	45.4817
KEGG_PATHWAY	hsa03040:Spliceosomes	19	2.753623188	8.04E-04	3304, 4670, 988, 57819, 4116, 3312, 51690, 9775, 1665, 3183, 9879, 1655, 3192, 56259, 3310, 3190, 7919, 9416, 11325	0.964501
KEGG_PATHWAY	hsa00190:Oxidative phosphorylation	19	2.753623188	0.001167	506, 9377, 1350, 1329, 509, 1327, 27068, 9551, 498, 522, 521, 6389, 5464, 10476, 10632, 4513, 539, 1347, 515	1.397273
KEGG_PATHWAY	hsa00010:Glycolysis / Gluconeogenesis	18	2.608695652	8.08E-08	3098, 128, 223, 226, 1738, 10327, 501, 5213, 5211, 3939, 2597, 2023, 2026, 5315, 2203, 5214, 3945, 2821	9.73E-05
KEGG_PATHWAY	hsa04666:Fc gamma R-mediated phagocytosis	17	2.463768116	2.40E-04	10059, 2934, 1785, 382, 1073, 1072, 10093, 10092, 4082, 6198, 1399, 10552, 10095, 10094, 10109, 998, 5594	0.288696
KEGG_PATHWAY	hsa05012:Parkinson's disease	17	2.463768116	0.006091	506, 9377, 1350, 1329, 509, 293, 1327, 292, 498, 522, 54205, 6389, 10476, 4513, 539, 1347, 515	7.096551
KEGG_PATHWAY	hsa04110:Cell cycle	16	2.31884058	0.011243	4173, 10971, 4172, 4175, 2810, 4174, 4176, 1029, 4171, 983, 9184, 7534, 3065, 7533, 7532, 7531	12.73646
KEGG_PATHWAY	hsa00480:Glutathione metabolism	14	2.028985507	8.09E-06	2936, 2937, 9446, 51056, 290, 4257, 373156, 2950, 2539, 3418, 79017, 3417, 2947, 5226	0.009742
KEGG_PATHWAY	hsa05130:Pathogenic Escherichia coli infection	14	2.028985507	3.73E-05	10971, 3875, 10093, 10092, 7430, 3688, 10552, 10095, 10094, 7534, 60, 10109, 998, 999	0.044956
KEGG_PATHWAY	hsa04142:Lysosomes	13	1.884057971	0.062791	8546, 967, 2799, 1522, 164, 1211, 1212, 1213, 3920, 1509, 3074, 1508, 3073	54.22146
KEGG_PATHWAY	hsa04722:Neurotrophin signaling pathway	13	1.884057971	0.088682	10971, 817, 581, 1399, 2885, 396, 7534, 7533, 808, 7532, 7531, 998, 5594	67.33631

KEGG_PATHWAY	hsa00620:Pyruvate metabolism	12	1.739130435	2.29E-05	4199, 223, 3939, 9380, 1738, 4191, 231, 4190, 3029, 5315, 501, 3945	0.027595
KEGG_PATHWAY	hsa00520:Amino sugar and nucleotide sugar metabolism	11	1.594202899	3.07E-04	64841, 3098, 2584, 2673, 10007, 5238, 3074, 29926, 2821, 3073, 29925	0.369145
KEGG_PATHWAY	hsa04612:Antigen processing and presentation	11	1.594202899	0.034257	811, 3304, 3320, 3107, 3310, 3312, 3309, 3326, 821, 3308, 1508	34.29519
KEGG_PATHWAY	hsa05322:Systemic lupus erythematosus	11	1.594202899	0.091654	718, 85235, 8968, 9555, 720, 85236, 81, 8359, 3015, 6741, 87	68.59685
KEGG_PATHWAY	hsa00020:Citrate cycle (TCA cycle)	10	1.449275362	8.10E-05	6389, 47, 3419, 1431, 3418, 1738, 3417, 4191, 4190, 50	0.097603
KEGG_PATHWAY	hsa00051:Fructose and mannose metabolism	10	1.449275362	1.78E-04	3098, 226, 231, 29926, 5214, 6652, 2203, 5213, 29925, 5211	0.2145
KEGG_PATHWAY	hsa00071:Fatty acid metabolism	10	1.449275362	6.64E-04	3033, 1892, 3032, 128, 223, 1374, 3030, 1632, 501, 37	0.797312
KEGG_PATHWAY	hsa05412:Arrhythmogenic right ventricular cardiomyopathy (ARVC)	10	1.449275362	0.047634	1832, 3688, 1674, 2010, 81, 60, 87, 1495, 4000, 488	44.45962
KEGG_PATHWAY	hsa04520:Adherens junction	10	1.449275362	0.051093	1457, 8826, 1460, 81, 60, 87, 1495, 998, 999, 5594	46.84186
KEGG_PATHWAY	hsa04260:Cardiac muscle contraction	10	1.449275362	0.054713	9377, 1350, 1329, 483, 481, 4513, 1327, 476, 1347, 488	49.23455
KEGG_PATHWAY	hsa00030:Pentose phosphate pathway	9	1.304347826	9.47E-05	2539, 226, 25796, 5214, 2203, 5213, 2821, 5211, 5226	0.114025
KEGG_PATHWAY	hsa00280:Valine, leucine and isoleucine degradation	9	1.304347826	0.005436	3033, 1892, 3032, 223, 3030, 1738, 64087, 501, 3028	6.356922
KEGG_PATHWAY	hsa00330:Arginine and proline metabolism	9	1.304347826	0.016641	445, 223, 2806, 1152, 51056, 2746, 501, 2744, 2805	18.3065
KEGG_PATHWAY	hsa00270:Cysteine and methionine metabolism	8	1.15942029	0.004496	3939, 27430, 58478, 2806, 4144, 1786, 3945, 2805	5.284791
KEGG_PATHWAY	hsa00250:Alanine, aspartate and glutamate metabolism	7	1.014492754	0.011425	445, 440, 2673, 2806, 2746, 2744, 2805	12.92986
KEGG_PATHWAY	hsa03030:DNA replication	7	1.014492754	0.023228	4173, 4172, 4175, 4174, 2237, 4176, 4171	24.66083
KEGG_PATHWAY	hsa00630:Glyoxylate and dicarboxylate metabolism	6	0.869565217	0.001699	4522, 1431, 9380, 4191, 4190, 50	2.027951
KEGG_PATHWAY	hsa00052:Galactose metabolism	6	0.869565217	0.021048	3098, 2584, 231, 5214, 5213, 5211	22.61049
KEGG_PATHWAY	hsa00640:Propanoate metabolism	6	0.869565217	0.047451	1892, 223, 3939, 3030, 501, 3945	44.33057
KEGG_PATHWAY	hsa00062:Fatty acid elongation in	4	0.579710145	0.010904	3033, 1892, 3032, 3030	12.37453

	mitochondria					
KEGG_PATHWAY	hsa00903:Limonene and pinene degradation	4	0.579710145	0.053548	1892, 223, 3030, 501	48.47577

Table S2 | Eighty differentially expressed proteins were identified in MCF-7/ADR compared to MCF-7.

Uniprot accession no.	Protein Description	Coverage	# Unique Peptides	Molecular Function	Cellular Component	Biological Process	Gene IDs	127/126	MW [kDa]	calc. pI
P05787	Keratin, type II cytoskeletal 8 OS=Homo sapiens GN=KRT8 PE=1 SV=7 - [K2C8_HUMAN]	56.52	28	catalytic activity; motor activity; structural molecule activity; protein binding	nucleus; organelle lumen; cytoplasm; cytoskeleton; membrane	cell death; cell organization and biogenesis; cell communication; regulation of biological process; response to stimulus; cell differentiation; development	3856	0.52	53.7	5.59
Q14315-2	Isoform 2 of Filamin-C OS=Homo sapiens GN=FLNC - [FLNC_HUMAN]	31.28	51	protein binding	cytoplasm; cytosol; cytoskeleton; membrane	cell organization and biogenesis; cell differentiation	2318	1.73	287.1	5.97
P07355	Annexin A2 OS=Homo sapiens GN=ANXA2 PE=1 SV=2 - [ANXA2_HUMAN]	56.05	20	enzyme regulator activity; metal ion binding; protein binding	extracellular; cytoplasm; endosome; membrane	development; metabolic process; regulation of biological process; transport; cell organization and biogenesis; coagulation; response to stimulus; cell proliferation	302	1.907	38.6	7.75
P04083	Annexin A1 OS=Homo sapiens GN=ANXA1 PE=1 SV=2 - [ANXA1_HUMAN]	60.69	17	DNA binding; RNA binding; enzyme regulator activity; protein binding; structural molecule activity;	cytoskeleton; extracellular; nucleus; cytoplasm; membrane; mitochondrion	defense response; regulation of biological process; response to stimulus; cellular component movement; cell communication; metabolic process; transport; cell differentiation; development; cell organization and biogenesis; cell death; cell proliferation; reproduction	301	1.501	38.7	7.02

				metal ion binding						
P04075	Fructose-bisphosphate aldolase A OS=Homo sapiens GN=ALDOA PE=1 SV=2 - [ALDOA_HUMAN]	48.9	18	protein binding; catalytic activity	extracellular; cytoplasm; cytosol; cytoskeleton; organelle lumen	transport; metabolic process; cell organization and biogenesis; coagulation; response to stimulus; regulation of biological process; cellular homeostasis	226	0.612	39.4	8.09
P08758	Annexin A5 OS=Homo sapiens GN=ANXA5 PE=1 SV=2 - [ANXA5_HUMAN]	71.25	20	enzyme regulator activity; metal ion binding; protein binding	cytoplasm; cell surface; membrane; extracellular	cell communication; regulation of biological process; response to stimulus; coagulation; cell death; metabolic process; cell organization and biogenesis	308	1.554	35.9	5.05
P08727	Keratin, type I cytoskeletal 19 OS=Homo sapiens GN=KRT19 PE=1 SV=4 - [K1C19_HUMAN]	51.75	17	structural molecule activity; protein binding	cytoskeleton; membrane; cytoplasm	response to stimulus; cell differentiation; cell organization and biogenesis; development	3880	0.414	44.1	5.14
P08183	Multidrug resistance protein 1 OS=Homo sapiens GN=ABCB1 PE=1 SV=3 - [MDR1_HUMAN]	26.33	30	nucleotide binding; transporter activity; protein binding; catalytic activity	cytoplasm; Golgi; membrane; cell surface	metabolic process; transport; response to stimulus; cell proliferation	5243	1.915	141.4	9
P02768	Serum albumin OS=Homo sapiens GN=ALB PE=1 SV=2 - [ALBU_HUMAN]	34.81	20	DNA binding; metal ion binding; protein binding; antioxidant	extracellular; cytoplasm; organelle lumen	transport; metabolic process; response to stimulus; coagulation; cell communication; cell death; regulation of biological process	213	2.017	69.3	6.28

				activity; catalytic activity						
P02647	Apolipoprotein A-I OS=Homo sapiens GN=APOA1 PE=1 SV=1 - [APOA1_HUMAN]	49.81	13	transporter activity; protein binding; enzyme reglator activity	extracellular; nucleus; cytoplasm; endosome; endoplasmic reticulum; organelle lumen; cytosol; membrane	metabolic process; regulation of biological process; cell proliferation; transport; response to stimulus; cell communication; coagulation; cell organization and biogenesis; cell differentiation; development; cellular component movement; defense response	335	1.864	30.8	5.76
P04792	Heat shock protein beta-1 OS=Homo sapiens GN=HSPB1 PE=1 SV=2 - [HSPB1_HUMAN]	43.9	9	protein binding; enzyme reglator activity	proteasome; nucleus; cytoplasm; cytoskeleton; cytosol; membrane; cell surface	development; metabolic process; regulation of biological process; cellular component movement; response to stimulus; cell death; cell communication	3315	0.551	22.8	6.4
P08729	Keratin, type II cytoskeletal 7 OS=Homo sapiens GN=KRT7 PE=1 SV=5 - [K2C7_HUMAN]	39.87	16	structural molecule activity; protein binding	nucleus; organelle lumen; cytoplasm; Golgi; cytoskeleton	metabolic process; regulation of biological process; cell organization and biogenesis	3855	1.844	51.4	5.48
P09211	Glutathione S-transferase P OS=Homo sapiens GN=GSTP1 PE=1 SV=2 - [GSTP1_HUMAN]	52.86	8	catalytic activity; protein binding; enzyme regulator activity	nucleus; cytoplasm; mitochondrion; cytosol	response to stimulus; defense response; regulation of biological process; metabolic process; development; cell communication; cell proliferation; cell death	2950	1.612	23.3	5.64
P06744	Glucose-6-phosphate isomerase OS=Homo sapiens GN=GPI	23.3	12	catalytic activity; protein binding	extracellular; nucleus; organelle lumen; cytoplasm; cytosol; membrane	development; metabolic process; response to stimulus; cell death; regulation of biological process	2821	0.667	63.1	8.32

	PE=1 SV=4 - [G6PI_HUMAN]									
P07339	Cathepsin D OS=Homo sapiens GN=CTSD PE=1 SV=1 - [CATD_HUMAN]	34.47	11	catalytic activity; protein binding	extracellular; cytoplasm; mitochondrion; vacuole; organelle lumen	cell communication; cell organization and biogenesis; metabolic process; response to stimulus; cell death	1509	0.523	44.5	6.54
P16402	Histone H1.3 OS=Homo sapiens GN=HIST1H1D PE=1 SV=2 - [H13_HUMAN]	32.58	3	DNA binding	chromosome; nucleus; organelle lumen	cell organization and biogenesis; metabolic process	3007	0.456	22.3	11.02
P07195	L-lactate dehydrogenase B chain OS=Homo sapiens GN=LDHB PE=1 SV=2 - [LDHB_HUMAN]	40.12	10	catalytic activity; protein binding; nucleotide binding	cytoplasm; mitochondrion; cytosol	metabolic process	3945	2.311	36.6	6.05
P11413	Glucose-6-phosphate 1-dehydrogenase OS=Homo sapiens GN=G6PD PE=1 SV=4 - [G6PD_HUMAN]	31.46	15	catalytic activity; protein binding; nucleotide binding	nucleus; cytoplasm; cytoskeleton; cytosol; membrane	metabolic process; regulation of biological process; response to stimulus; cell differentiation; development; cell death	2539	0.498	59.2	6.84
P08133-2	Isoform 2 of Annexin A6 OS=Homo sapiens GN=ANXA6 - [ANXA6_HUMAN]	33.39	20	metal ion binding; protein binding	membrane; cytoplasm; extracellular	transport; regulation of biological process	309	1.501	72.4	5.6
P48735	Isocitrate	40.49	15	metal ion	cytoplasm;	metabolic process	3418	0.643	50.9	8.69

	dehydrogenase [NADP], mitochondrial OS=Homo sapiens GN=IDH2 PE=1 SV=2 - [IDHP_HUMAN]			binding; catalytic activity; nucleotide binding	mitochondrion; membrane; organelle lumen					
Q92688-2	Isoform 2 of Acidic leucine-rich nuclear phosphoprotein 32 family member B OS=Homo sapiens GN=ANP32B - [AN32B_HUMAN]	37.44	3		nucleus; cytoplasm		10541	0.627	22.3	4.3
P00738	Haptoglobin OS=Homo sapiens GN=HP PE=1 SV=1 - [HPT_HUMAN]	27.09	11	catalytic activity; protein binding; antioxidant activity	extracellular; cytoplasm; organelle lumen	metabolic process; cellular homeostasis; defense response; response to stimulus; cell death; regulation of biological process	3240	2.277	45.2	6.58
Q01469	Fatty acid-binding protein, epidermal OS=Homo sapiens GN=FABP5 PE=1 SV=3 - [FABP5_HUMAN]	64.44	7	transporter activity; protein binding	cytoplasm	metabolic process; transport; development; response to stimulus	2171	2.122	15.2	7.01
P01023	Alpha-2-macroglobulin OS=Homo sapiens	11.26	14	protein binding; enzyme regulator	extracellular; cytoplasm; cytosol; organelle lumen	defense response; metabolic process; regulation of biological process; response to stimulus; transport; cell	2	1.651	163.2	6.46

	GN=A2M PE=1 SV=3 - [A2MG_HUMAN]			activity		communication; coagulation; cell differentiation				
P01024	Complement C3 OS=Homo sapiens GN=C3 PE=1 SV=2 - [CO3_HUMAN]	13.53	20	enzyme regulator activity; protein binding	extracellular; membrane	defense response; regulation of biological process; response to stimulus; metabolic process; cell communication; transport; development; cell organization and biogenesis	718	1.518	187	6.4
P01857	Ig gamma-1 chain C region OS=Homo sapiens GN=IGHG1 PE=1 SV=1 - [IGHG1_HUMAN]	32.42	7	protein binding	extracellular; membrane	response to stimulus; metabolic process; regulation of biological process; cell communication; transport; defense response	3500	1.598	36.1	8.19
P00918	Carbonic anhydrase 2 OS=Homo sapiens GN=CA2 PE=1 SV=2 - [CAH2_HUMAN]	33.85	7	catalytic activity; protein binding; metal ion binding	extracellular; cytoplasm; cytosol; membrane	development; metabolic process; response to stimulus; transport; cellular homeostasis; regulation of biological process; cell communication; cell differentiation	760	0.545	29.2	7.4
P01009	Alpha-1-antitrypsin OS=Homo sapiens GN=SERPINA1 PE=1 SV=3 - [A1AT_HUMAN]	21.77	9	protein binding; enzyme regulator activity	extracellular; cytoplasm; endoplasmic reticulum; organelle lumen	response to stimulus; transport; defense response; coagulation; metabolic process; regulation of biological process	5265	1.896	46.7	5.59
O75874	Isocitrate dehydrogenase [NADP] cytoplasmic OS=Homo sapiens GN=IDH1 PE=1 SV=2 -	28.5	10	metal ion binding; catalytic activity; protein binding; nucleotide binding	cytoplasm; mitochondrion; organelle lumen; cytosol	metabolic process; response to stimulus; development; reproduction	3417	0.568	46.6	7.01

	[IDHC_HUMAN]									
Q13185	Chromobox protein homolog 3 OS=Homo sapiens GN=CBX3 PE=1 SV=4 - [CBX3_HUMAN]	42.08	6	protein binding	chromosome; nucleus; membrane; organelle lumen; cytoskeleton	cell organization and biogenesis; metabolic process; regulation of biological process	11335	0.642	20.8	5.33
P17931	Galectin-3 OS=Homo sapiens GN=LGALS3 PE=1 SV=5 - [LEG3_HUMAN]	28.4	6	protein binding	extracellular; nucleus; spliceosomal complex; cytoplasm; membrane; mitochondrion	development; metabolic process; cell differentiation; cell organization and biogenesis; defense response; response to stimulus	3958	0.657	26.1	8.56
P09467	Fructose-1,6-bisphosphatase 1 OS=Homo sapiens GN=FBP1 PE=1 SV=5 - [F16P1_HUMAN]	27.22	8	catalytic activity; protein binding; nucleotide binding; metal ion binding	cytoplasm; cytosol	metabolic process; regulation of biological process; cell growth; cell organization and biogenesis; response to stimulus; cell communication	2203	0.668	36.8	6.99
Q9Y696	Chloride intracellular channel protein 4 OS=Homo sapiens GN=CLIC4 PE=1 SV=4 - [CLIC4_HUMAN]	41.5	8	transporter activity; protein binding	nucleus; cytoplasm; mitochondrion; vacuole; cytoskeleton; cytosol; membrane; cell surface; organelle lumen	development; cell differentiation; cell organization and biogenesis; transport; cellular homeostasis; reproduction; cellular component movement; regulation of biological process; response to stimulus	25932	1.679	28.8	5.59
P51572	B-cell receptor-associated protein 31 OS=Homo sapiens GN=BCAP31 PE=1 SV=3 -	32.11	9	protein binding	cytoplasm; Golgi; membrane; endoplasmic reticulum; cytosol	transport; cell death; cell organization and biogenesis; response to stimulus; cellular homeostasis; reproduction; cell communication; regulation of biological process; metabolic process	10134	0.547	28	8.44

	[BAP31_HUMAN]									
P68871	Hemoglobin subunit beta OS=Homo sapiens GN=HBB PE=1 SV=2 - [HBB_HUMAN]	57.14	7	antioxidant activity; catalytic activity; transporter activity; metal ion binding; protein binding	extracellular; cytoplasm; cytosol; organelle lumen	transport; coagulation; response to stimulus; cell death; regulation of biological process; metabolic process; cell organization and biogenesis	3043	1.516	16	7.28
P15121	Aldose reductase OS=Homo sapiens GN=AKR1B1 PE=1 SV=3 - [ALDR_HUMAN]	17.09	4	catalytic activity	extracellular; nucleus; organelle lumen; cytoplasm; cytosol	metabolic process; response to stimulus	231	1.841	35.8	6.98
P0CG05	Ig lambda-2 chain C regions OS=Homo sapiens GN=IGLC2 PE=1 SV=1 - [LAC2_HUMAN]	48.11	4	protein binding	extracellular; membrane	metabolic process; regulation of biological process; response to stimulus; cell communication; transport; defense response	3538	1.618	11.3	7.24
Q9UHB6-2	Isoform Alpha of LIM domain and actin-binding protein 1 OS=Homo sapiens GN=LIMA1 - [LIMA1_HUMAN]	26.38	13	protein binding; metal ion binding	cytoskeleton; cytoplasm; membrane	cell organization and biogenesis; regulation of biological process	51474	1.748	67.1	5.88
Q96CN7	Isochorismatase domain-containing protein 1 OS=Homo sapiens	19.46	5	catalytic activity	cytoplasm	metabolic process	51015	0.613	32.2	7.39

	GN=ISOC1 PE=1 SV=3 - [ISOC1_HUMAN]									
P02765	Alpha-2-HS-glycoprotein OS=Homo sapiens GN=AHSG PE=1 SV=1 - [FETUA_HUMAN]	15.53	6	enzyme regulator activity	extracellular	development; transport; defense response; response to stimulus; reproduction; metabolic process; regulation of biological process; cell growth; cell organization and biogenesis; cell communication	197	2.102	39.3	5.72
P16070-18	Isoform 18 of CD44 antigen OS=Homo sapiens GN=CD44 - [CD44_HUMAN]	18.82	6	catalytic activity; receptor activity; signal transducer activity; protein binding	membrane; cell surface	development; defense response; response to stimulus; metabolic process; regulation of biological process; cell communication; cell organization and biogenesis; cell death; reproduction	960	1.936	37.3	5.52
P69905	Hemoglobin subunit alpha OS=Homo sapiens GN=HBA1 PE=1 SV=2 - [HBA_HUMAN]	25.35	3	antioxidant activity; catalytic activity; transporter activity; metal ion binding; protein binding	extracellular; cytoplasm; cytosol; ribosome; organelle lumen	transport; cell death; regulation of biological process; response to stimulus; metabolic process; cell organization and biogenesis	3040	2.291	15.2	8.68
P17096-2	Isoform HMG-Y of High mobility group protein HMG-I/HMG-Y OS=Homo sapiens GN=HMGA1 - [HMGA1_HUMAN]	17.71	2	DNA binding; catalytic activity; protein binding	chromosome; nucleus; organelle lumen; cytoplasm; cytosol	metabolic process; response to stimulus; cell organization and biogenesis; regulation of biological process; cell proliferation	3159	3.484	10.7	10.32
P01876	Ig alpha-1 chain	16.43	5	protein	extracellular	response to stimulus; metabolic	3493	2.478	37.6	6.51

	C region OS=Homo sapiens GN=IGHA1 PE=1 SV=2 - [IGHA1_HUMAN]			binding		process				
Q8TD06	Anterior gradient protein 3 homolog OS=Homo sapiens GN=AGR3 PE=1 SV=1 - [AGR3_HUMAN]	12.65	2	protein binding	extracellular		155465	0.449	19.2	7.99
Q9Y2T3	Guanine deaminase OS=Homo sapiens GN=GDA PE=1 SV=1 - [GUAD_HUMAN]	10.13	5	metal ion binding; catalytic activity	cytoplasm; cytosol	metabolic process; development	9615	1.852	51	5.68
O75223	Gamma-glutamylcyclotransferase OS=Homo sapiens GN=GGCT PE=1 SV=1 - [GGCT_HUMAN]	21.28	4	catalytic activity; protein binding	cytoplasm; cytosol	cell communication; cell death; cell organization and biogenesis; regulation of biological process; response to stimulus; metabolic process	79017	0.631	21	5.14
P17655	Calpain-2 catalytic subunit OS=Homo sapiens GN=CAPN2 PE=1 SV=6 - [CAN2_HUMAN]	12.43	8	catalytic activity; metal ion binding; protein binding	chromosome; nucleus; cytoplasm; membrane	response to stimulus; development; metabolic process; cell differentiation	824	1.568	79.9	4.98
Q9Y6M1	Isoform 2 of	11.69	5	nucleotide	nucleus; cytoplasm;	metabolic process; regulation	10644	1.535	61.8	8.25

-1	Insulin-like growth factor 2 mRNA-binding protein 2 OS=Homo sapiens GN=IGF2BP2 - [IF2B2_HUMAN]			binding; RNA binding; protein binding; translation regulator activity	cytosol; cytoskeleton	of biological process; transport				
P02656	Apolipoprotein C-III OS=Homo sapiens GN=APOC3 PE=1 SV=1 - [APOC3_HUMAN]	27.27	2	enzyme regulator activity; protein binding	extracellular; cytoplasm; endosome	metabolic process; transport; defense response; response to stimulus; cell communication; regulation of biological process; cell organization and biogenesis; cellular homeostasis	345	1.874	10.8	5.41
P17661	Desmin OS=Homo sapiens GN=DES PE=1 SV=3 - [DESM_HUMAN]	5.53	2	structural molecule activity; protein binding	cytoplasm; cytosol; cytoskeleton; membrane	cell organization and biogenesis; regulation of biological process; cellular component movement	1674	1.692	53.5	5.27
P05114	Non-histone chromosomal protein HMG-14 OS=Homo sapiens GN=HMGN1 PE=1 SV=3 - [HMGN1_HUMAN]	40	4	DNA binding; protein binding	chromosome; nucleus; cytoplasm	metabolic process; response to stimulus; cell organization and biogenesis; regulation of biological process; development; cell proliferation	3150	0.588	10.7	9.6
P02652	Apolipoprotein A-II OS=Homo sapiens GN=APOA2 PE=1 SV=1 - [APOA2_HUMAN]	19	3	transporter activity; protein binding; enzyme regulator activity	extracellular; cytoplasm; endosome; endoplasmic reticulum; organelle lumen; cytosol	metabolic process; defense response; response to stimulus; regulation of biological process; transport; cell communication; cell organization and biogenesis; development	336	2.141	11.2	6.62
P13473	Lysosome-associated membrane	4.88	2		cytoplasm; vacuole; membrane; endosome	transport; coagulation; response to stimulus	3920	0.431	44.9	5.63

	glycoprotein 2 OS=Homo sapiens GN=LAMP2 PE=1 SV=2 - [LAMP2_HUMAN]									
P45973	Chromobox protein homolog 5 OS=Homo sapiens GN=CBX5 PE=1 SV=1 - [CBX5_HUMAN]	25.65	3	protein binding	nucleus; organelle lumen; chromosome; cytoplasm	coagulation; response to stimulus; metabolic process; regulation of biological process	23468	0.542	22.2	5.86
Q9BS40	Latexin OS=Homo sapiens GN=LXN PE=1 SV=2 - [LXN_HUMAN]	13.96	3	enzyme regulator activity	cytoplasm	defense response; response to stimulus; metabolic process; regulation of biological process	56925	0.37	25.7	5.78
P08243-3	Isoform 3 of Asparagine synthetase [glutamine-hydrolyzing] OS=Homo sapiens GN=ASNS - [ASNS_HUMAN]	3.97	2	catalytic activity; nucleotide binding; protein binding	cytoplasm; cytosol; membrane	development; metabolic process; cell communication; regulation of biological process; response to stimulus; cell death	440	0.654	54.8	6.49
Q13642-1	Isoform 1 of Four and a half LIM domains protein 1 OS=Homo sapiens GN=FHL1 - [FHL1_HUMAN]	12.14	3	protein binding; metal ion binding	nucleus; cytoplasm; cytosol; membrane	regulation of biological process; development; cell differentiation; cell growth; cell organization and biogenesis; transport	2273	1.644	31.9	8.32
Q02952-3	Isoform 3 of	3.76	7	protein	cytoplasm;	transport; cell communication;	9590	2.298	180.9	4.44

	A-kinase anchor protein 12 OS=Homo sapiens GN=AKAP12 - [AKA12_HUMAN]			binding	cytoskeleton; membrane	regulation of biological process; response to stimulus; metabolic process				
P16422	Epithelial cell adhesion molecule OS=Homo sapiens GN=EPCAM PE=1 SV=2 - [EPCAM_HUMAN]	8.6	2	protein binding	membrane; cell surface	development; cell proliferation; regulation of biological process	4072	0.616	34.9	7.46
Q9ULA0	Aspartyl aminopeptidase OS=Homo sapiens GN=DNPEP PE=1 SV=1 - [DNPEP_HUMAN]	3.79	2	catalytic activity; metal ion binding	cytoplasm	metabolic process	23549	0.67	52.4	7.42
Q96AY3	Peptidyl-prolyl cis-trans isomerase FKBP10 OS=Homo sapiens GN=FKBP10 PE=1 SV=1 - [FKB10_HUMAN]	7.22	4	catalytic activity; metal ion binding	cytoplasm; endoplasmic reticulum; organelle lumen; membrane	metabolic process	60681	1.529	64.2	5.62
O43768-2	Isoform 2 of Alpha-endosulfine OS=Homo sapiens GN=ENSA - [ENSA_HUMAN]	27.35	3	enzyme regulator activity; protein binding	nucleus; organelle lumen; cytoplasm	transport; cell division; cell organization and biogenesis; response to stimulus	2029	0.655	13	8.27

Q6UXG2-3	Isoform 3 of UPF0577 protein KIAA1324 OS=Homo sapiens GN=KIAA1324 - [K1324_HUMAN]	4.54	3				57535	0.627	101.9	6.55
P08962-3	Isoform 3 of CD63 antigen OS=Homo sapiens GN=CD63 - [CD63_HUMAN]	11.54	2	protein binding	cytoplasm; membrane; vacuole; cell surface; endosome	transport; coagulation; response to stimulus; cellular component movement; regulation of biological process; cell differentiation; cell organization and biogenesis	967	0.564	17.3	7.88
P17516	Aldo-keto reductase family 1 member C4 OS=Homo sapiens GN=AKR1C4 PE=1 SV=3 - [AK1C4_HUMAN]	4.64	2	catalytic activity; transporter activity	cytoplasm; cytosol	metabolic process; transport; cell communication; regulation of biological process; response to stimulus	1109	0.523	37	6.93
Q9NQW6-2	Isoform 2 of Actin-binding protein anillin OS=Homo sapiens GN=ANLN - [ANLN_HUMAN]	1.66	2	protein binding	nucleus; cytoplasm; cytoskeleton	cell division; cell organization and biogenesis; regulation of biological process	54443	1.529	119.9	8.16
Q12841	Follistatin-related protein 1 OS=Homo sapiens GN=FSTL1 PE=1 SV=1 - [FSTL1_HUMAN]	11.36	4	metal ion binding; protein binding	extracellular	cell communication; metabolic process; regulation of biological process; response to stimulus	11167	2.29	35	5.52

Q9UN81	LINE-1 retrotransposable element ORF1 protein OS=Homo sapiens GN=L1RE1 PE=1 SV=1 - [LORF1_HUMAN]	6.51	2	nucleotide binding; DNA binding; RNA binding; structural molecule activity; protein binding	cytoplasm; nucleus; organelle lumen; vacuole; endoplasmic reticulum		4029	0.587	40	9.51
P14735-2	Isoform 2 of Insulin-degrading enzyme OS=Homo sapiens GN=IDE - [IDE_HUMAN]	4.74	2	protein binding; catalytic activity; nucleotide binding; metal ion binding	extracellular; nucleus; cytoplasm; mitochondrion; organelle lumen; cytosol; membrane; cell surface; proteasome	metabolic process; cell communication; regulation of biological process; response to stimulus; development; cellular homeostasis; cell organization and biogenesis	3416	0.668	54.2	6.71
Q05682-5	Isoform 5 of Caldesmon OS=Homo sapiens GN=CALD1 - [CALD1_HUMAN]	5.83	3	protein binding	cytoplasm; cytosol; cytoskeleton; membrane	cellular component movement; cell organization and biogenesis	800	1.915	61.2	6.71
P32320	Cytidine deaminase OS=Homo sapiens GN=CDA PE=1 SV=2 - [CDD_HUMAN]	25.34	2	catalytic activity; metal ion binding; protein binding	extracellular; cytoplasm; cytosol	metabolic process; cell communication; regulation of biological process; response to stimulus; cell growth; cell organization and biogenesis	978	1.904	16.2	6.92
P10909-4	Isoform 4 of Clusterin OS=Homo sapiens GN=CLU - [CLUS_HUMAN]	6.49	2	protein binding; catalytic activity	extracellular; nucleus; cytoplasm; mitochondrion; endoplasmic reticulum; cytosol; organelle lumen; membrane	cell communication; cell death; cell organization and biogenesis; regulation of biological process; response to stimulus; transport; metabolic process; coagulation; cell proliferation; development;	1191	1.529	48.8	6.71

						defense response; cell differentiation				
Q96DG6	Carboxymethylenebutenolidase homolog OS=Homo sapiens GN=CMBL PE=1 SV=1 - [CMBL_HUMAN]	12.24	3	catalytic activity	cytoplasm; cytosol	metabolic process	134147	0.372	28	7.18
Q15417	Calponin-3 OS=Homo sapiens GN=CNN3 PE=1 SV=1 - [CNN3_HUMAN]	6.38	2	protein binding	cytoskeleton	cell organization and biogenesis; metabolic process; regulation of biological process	1266	1.73	36.4	6.05
O95571	Persulfide dioxygenase ETHE1, mitochondrial OS=Homo sapiens GN=ETHE1 PE=1 SV=2 - [ETHE1_HUMAN]	8.27	2	metal ion binding; catalytic activity	nucleus; organelle lumen; cytoplasm; mitochondrion	metabolic process	23474	1.555	27.9	6.83
Q16643	Drebrin OS=Homo sapiens GN=DBN1 PE=1 SV=4 - [DREB_HUMAN]	4.31	2	protein binding	cytoplasm; membrane; cytoskeleton	cell organization and biogenesis; development; cell communication; cell differentiation; regulation of biological process; cell proliferation	1627	1.744	71.4	4.45
Q9UBR2	Cathepsin Z OS=Homo sapiens GN=CTSZ PE=1 SV=1 - [CATZ_HUMA	7.26	2	catalytic activity	extracellular; cytoplasm; vacuole; endoplasmic reticulum; membrane	metabolic process; regulation of biological process; development	1522	1.564	33.8	7.11

	N]								
P56159-2	Isoform 2 of GDNF family receptor alpha-1 OS=Homo sapiens GN=GFRA1 - [GFRA1_HUMAN]	8.04	4	receptor activity; protein binding; signal transducer activity	extracellular; membrane; cell surface	development; cell communication; regulation of biological process; response to stimulus; cell differentiation; cell organization and biogenesis; cellular component movement; reproduction	2674	0.662	50.8	7.99
Q17RY6	Lymphocyte antigen 6K OS=Homo sapiens GN=LY6K PE=1 SV=2 - [LY6K_HUMAN]	13.33	2		extracellular; nucleus; organelle lumen; cytoplasm; membrane		54742	1.825	18.7	7.43

Table S3 | DEPS were enriched within KEGG pathway.

**Term**	**Count**	**%**	**P**	**Genes Symbol**
Glutathione metabolism	5	6.33	*0.00027*	GGCT,G6PD,GSTP1, IDH1, IDH2
Pentose phosphate pathway	4	5.06	*0.00052*	ALDOA, FBP1, GPI, G6PD
Glycolysis / Gluconeogenesis	4	5.06	*0.00662*	ALDOA, FBP1,GPI,LDHB
PPAR signaling pathway	4	5.06	*0.00975*	APOA1, APOA2, APOC3, F ABP5
Fructose and mannose metabolism	3	3.80	*0.02013*	AKR1B1, ALDOA, FBP1
Lysosome	4	5.06	*0.03925*	CD63, CTSD, CTSZ, LAMP2
Complement and coagulation cascades	3	3.80	0.07319	A2M, SERPINA1,C3

Table S4 | Thirty-two modules were identified in network.

Module	Nodes	Edges	Gene symbol
2	20	49	DAXX, KRT18, CASP3, BCL2, CRYAA, HIPK2, TIAM1, HBEGF, CRYBB2, EGFR, CRYGC, BCL2L1, RET, HSPB1, BCAP31, CEACAM1, CRYAB, ERBB4, CD44, MAPK8
3	19	46	PRKCA, EZR, ROCK1, SRC, INSR, NR3C1, DAG1, NF2, MSN, KDR, SP1, HNF1A, CD46, PRKACA, SHC1, HLA-A, ADRBK1, HSPA1A, EP300
4	19	56	CDC42, TP53, LCK, SLC9A1, RAF1, FGFR3, PPARG, HBB, DLG4, -, CASP9, MDFI, HBG2, MAP2K4, EWSR1, YWHAG, FYN, TGFBR1, PPA1
12	17	36	AR, HIST3H3, TRPM7, RPS6KA4, HDAC5, MBD1, CSK, RPS6KA3, CREBBP, MAPK1, KRT8, SMAD1, CASP8, DMD, PLAT, CBX5, HMGN2
15	15	26	DNMBP, PRNP, A2M, DNM1, APOE, LEP, CTSE, NCAM1, HSPD1, GDNF, GFRA1, NGF, PDGFA, PDGFB, LEPR
1	8	16	USHBP1, KRT15, ABI2, HGS, EXOC8, DGCR6L, KRT6B, KRT19
8	7	10	C3, CFB, CFH, CFP, CFI, C2, MASP1
14	7	14	ABL1, SRF, GRB2, JUN, HMGA1, FHL2, RASA1
11	6	13	CALM1, ZHX1, CSNK2A1, RELA, KPNA2, SAT1
13	6	11	ALDOA, LGALS13, ACTG1, TUBB, HSPA8, DYNLL1
23	6	10	DLG1, APOH, PLG, TTR, PAPPA, LRP2
6	5	9	CBX1, HIST1H1E, CBX3, PIM1, CHAF1A
29	5	7	XPO1, PRKCB, ANXA2, PHB, MYOC
21	4	6	AGA, ALB, FCGRT, IGF2R
5	3	6	NSL1, MIS12, DSN1
7	3	3	HLA-DMB, HLA-DRA, CD63
9	3	4	CBX4, RING1, RNF2
10	3	4	TGFBI, FN1, COL2A1
22	3	6	CAV1, CEBPB, MAPK3
28	3	5	BIN1, FBP1, FXR2
30	3	5	PKN1, TRAF2, ACTN1
16	2	3	TRIM28, TRIM24
17	2	3	CPN1, C5
18	2	3	ELANE, SERPINA1

19	2	3	PTN, LUC7L2
20	2	3	TGFBR2, CDK1
24	2	3	SHBG, PLP2
25	2	3	S100A4, S100A1
26	2	3	C1orf103, GC
27	2	3	FURIN, LRP1
31	2	3	KNG1, ITGB2
32	2	3	INS, CTSB

Table S5 | Relation of DEPs-one step interacting proteins.

One step-protein	number	DEPs
SUMO4	5	GSTP1,G6PD,IDH1,ALDOA,LDHB
EGFR	4	ANXA1,KRT7,KRT8,CD44
PRKCA	4	AKAP12,FLNC,ANXA2,HMGN1
MYOC	4	A2M,LGALS3,ALDOA,ANXA2
APP	4	CLU,CTSD,IDE,BCAP31
LRP1	3	C3,SERPINA1,A2M
IGFBP3	3	CTSD,GPI,CD44
CSNK2A1	3	LGALS3,GPI,HMGA1
KLK3	3	SERPINA1,ALB,A2M
PON1	3	CLU,APOA1,ALB
SLC2A4	3	CTSD,FKBP10,IDH2
CTSB	3	ANXA2,CTSD,A2M
SHBG	3	CTSD,DES,A2M
TGM2	3	GSTP1,ALDOA,ANXA1
MMP2	2	LGALS3,A2M
MMP7	2	CD44,SERPINA1
GRB2	2	CALD1,ANXA2
CDK1	2	CALD1,HMGA1
CELA1	2	SERPINA1,A2M
HBB	2	HP,HBA2
DMD	2	KRT19,KRT8
LBR	2	CBX3,CBX5
PLTP	2	APOA2,APOA1

ELN	2	LGALS3,FKBP10
S100A1	2	ANXA6,DES
ITGAM	2	C3,HP
TGFBR2	2	CD44,CLU
DSN1	2	CBX3,CBX5
CUBN	2	ALB,LGALS3
YWHAZ	2	CLIC4,HMGN1
CBX1	2	CBX3,CBX5
S100B	2	ANXA6,DES
C1orf103	2	HSPB1,ETHE1
APOF	2	APOA2,APOA1
YWHAG	2	LIMA1,ALB
PLAT	2	KRT8,ANXA2
DYNLL1	2	CA2,ALDOA
MAPK8	2	GSTP1,KRT8
TRIM28	2	CBX3,CBX5
SRC	2	ANXA1,ANXA2
MKI67	2	CBX3,CBX5
ACTG1	2	ANXA5,BCAP31
TAF4	2	CBX5,CBX3
ATF7IP	2	KRT7,A2M
APOA1BP	2	APOA2,APOA1
LEP	2	A2M,CLU
HIST3H3	2	CBX5,CBX3
HIST1H1E	2	CBX5,CBX3
APOE	2	ALB,A2M

CSNK2A2	2	LGALS3,HMGA1
TRIM24	2	CBX3,CBX5
ADRBK1	2	CAPN2,AKAP12
MIS12	2	CBX3,CBX5
LCAT	2	APOA1,A2M
NFKBIA	2	HSPB1,CAPN2
CD4	2	CD44,CTSD
PKD1	2	DES,KRT8
XRCC6	2	CLU,CBX5
ADAMTS4	2	SERPINA1,HP
TGFBR1	2	CD44,CLU
SRGN	2	CD44,ALB
CTSG	2	C3,SERPINA1
SUFU	2	HMGA1,LGALS3
PNN	2	KRT19,KRT8
DYSF	2	ANXA1,ANXA2
ZNF598	2	DBN1,CDA
FYN	2	CNN3,CD44
AP4M1	2	LAMP2,CD63
IGSF21	2	HSPB1,ETHE1
INS	2	CTSD,IDE
LRP2	2	CLU,ALB
AMBP	2	ALB,A2M
EIF4G1	2	HSPB1,ANXA5
CRYAA	2	HSPB1,ALB
HSPA5	2	KRT8,A2M

CR2	2	ANXA6,C3
SRF	2	HMGA1,FHL1

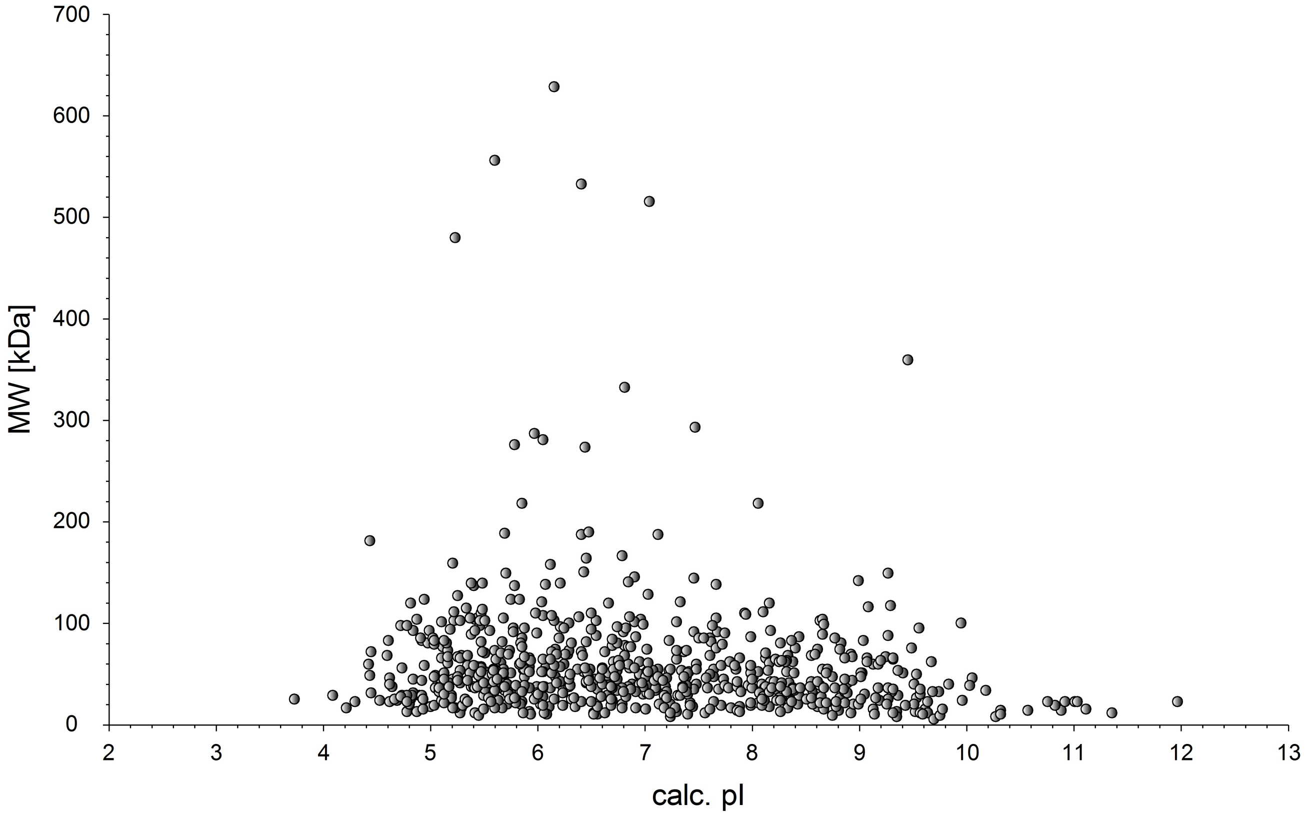

Figure S1 | The molecular weights (MW) of the identified proteins spanned mainly from 10,000 to 200,000 Da. Most proteins had an isoelectric point (pI) between 4 and 10.

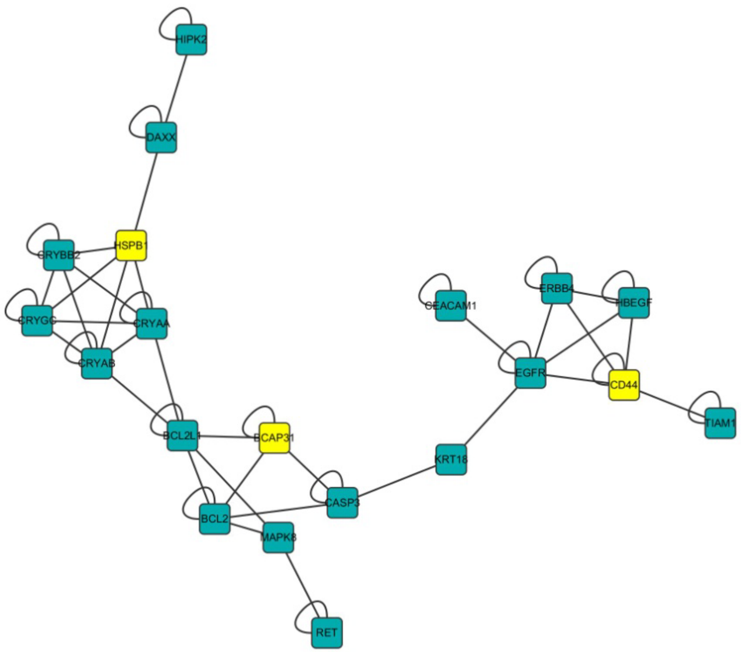

Figure S2 | Functional network of identified the 2nd module. This module contained 20 proteins which were enriched within 11 pathways, among them, there were six pathways associated with resistance, especially MAPK signal pathway. There were five proteins in MAPK signal pathway, including one pair of DEPs-one step interacting protein (HSPB1-DAXX).The yellow node indicates DEPs and the green node indicates one-step interacting protein.

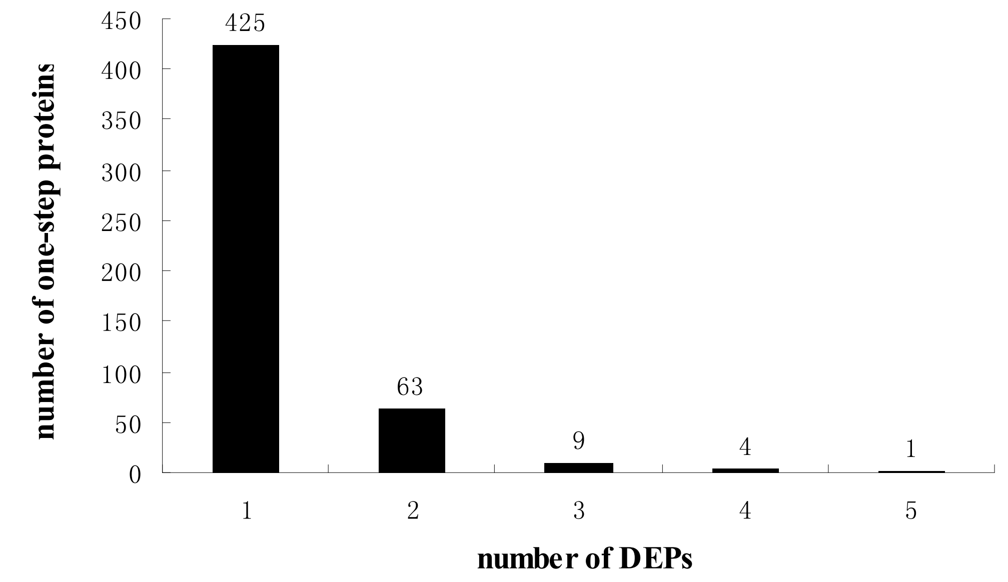

Figure S3 | DEPs-one step interacting protein relations. The number of DEPs interacting with one-step protein ranged from 1 to 5. It represented that 425 one-step proteins correlate with 1 DEPs, 63 one-step proteins correlate with 2 DEPs, 9 one-step proteins correlate with 3 DEPs, 4 one-step proteins correlate with 4 DEPs and 1 one-step protein correlate with 5 DEPs in the PPI.
